# Agomirs upregulating carboxypeptidase E expression rescue hippocampal neurogenesis and memory deficits in Alzheimer’s disease

**DOI:** 10.1186/s40035-024-00414-z

**Published:** 2024-04-26

**Authors:** Dongfang Jiang, Hongmei Liu, Tingting Li, Song Zhao, Keyan Yang, Fuwen Yao, Bo Zhou, Haiping Feng, Sijia Wang, Jiaqi Shen, Jinglan Tang, Yu-Xin Zhang, Yun Wang, Caixia Guo, Tie-Shan Tang

**Affiliations:** 1 Key Laboratory of Organ Regeneration and Reconstruction, State Key Laboratory of Membrane Biology, Institute of Zoology, Chinese Academy of Sciences, Beijing, 100101 China; 2 Beijing Institute of Genomics, Chinese Academy of Sciences/China National Center for Bioinformation, Beijing, 100101 China; 3 Beijing Institute for Stem Cell and Regenerative Medicine, Beijing, 100101 China; 4 University of Chinese Academy of Sciences, Chinese Academy of Sciences, Beijing, 100101 China; 5 Present Address: Department of Psychology, UC San Diego, La Jolla, CA 92093 USA

**Keywords:** Carboxypeptidase E, Adult hippocampal neurogenesis, Agomir, BDNF, Alzheimer’s disease, Memory deficit, Intranasal instillation

## Abstract

**Background:**

Adult neurogenesis occurs in the subventricular zone (SVZ) and the subgranular zone of the dentate gyrus in the hippocampus. The neuronal stem cells in these two neurogenic niches respond differently to various physiological and pathological stimuli. Recently, we have found that the decrement of carboxypeptidase E (CPE) with aging impairs the maturation of brain-derived neurotrophic factor (BDNF) and neurogenesis in the SVZ. However, it remains unknown whether these events occur in the hippocampus, and what the role of CPE is in the adult hippocampal neurogenesis in the context of Alzheimer’s disease (AD).

**Methods:**

In vivo screening was performed to search for miRNA mimics capable of upregulating CPE expression and promoting neurogenesis in both neurogenic niches. Among these, two agomirs were further assessed for their effects on hippocampal neurogenesis in the context of AD. We also explored whether these two agomirs could ameliorate behavioral symptoms and AD pathology in mice, using direct intracerebroventricular injection or by non-invasive intranasal instillation.

**Results:**

Restoration of CPE expression in the hippocampus improved BDNF maturation and boosted adult hippocampal neurogenesis. By screening the miRNA mimics targeting the 5’UTR region of *Cpe* gene, we developed two agomirs that were capable of upregulating CPE expression. The two agomirs significantly rescued adult neurogenesis and cognition, showing multiple beneficial effects against the AD-associated pathologies in APP/PS1 mice. Of note, noninvasive approach via intranasal delivery of these agomirs improved the behavioral and neurocognitive functions of APP/PS1 mice.

**Conclusions:**

CPE may regulate adult hippocampal neurogenesis via the CPE–BDNF–TrkB signaling pathway. This study supports the prospect of developing miRNA agomirs targeting CPE as biopharmaceuticals to counteract aging- and disease-related neurological decline in human brains.

**Supplementary Information:**

The online version contains supplementary material available at 10.1186/s40035-024-00414-z.

## Background

The mammalian hippocampal dentate gyrus (DG) is a niche for adult neurogenesis from neural stem cells (NSCs) and is involved in learning and memory. Newly formed neurons can integrate into the circuitry of the adult hippocampus. Hippocampal neurogenesis occurs during development and into adulthood. Aging causes a steady decline of neurogenesis [1, 2], which could be exacerbated in pathological conditions such as Alzheimer’s disease (AD) [3, 4]. Impaired adult neurogenesis leads to the loss of different neurons and reduction of cell renewal capacity in the adult brain as well as the putative functions of these new neurons. Some symptoms observed in the early stages of AD, such as cognitive impairment, may be connected to dysfunctions of adult neurogenesis. Compelling evidence shows that there is decreased hippocampal neurogenesis in different animal models of AD [5], and that the anti-diabetic drug rosiglitazone prevents stress-related depression through enhancement of adult neurogenesis [6]. Therefore, NSCs could be a potential target for the treatment of neurodegenerative diseases.

Recently, using ligand-receptor interaction analysis, our single-cell transcriptomic data show that the expression of certain receptor genes, such as *Ntrk2* (encoding TrkB), is a crucial step in the mobilization of quiescent NSCs during neurogenesis. Downregulation of the TrkB pathway has been observed in old NSCs in both the subventricular zone (SVZ) and DG [7]. Therefore, deficiency of the BDNF-TrkB cascade could be a causative factor for the age-induced adult neurogenesis reduction in both neurogenic niches. Furthermore, we found that diminishment of carboxypeptidase E (CPE) in the SVZ with aging results in impaired maturation of BDNF, which limits neurogenesis in the SVZ [7]. CPE is a prohormone- and proneuropeptide-processing exopeptidase that plays diverse roles in prohormone sorting, protein internalization, regulation of signaling pathway and neuroprotection [6, 8–11]. Given that the NSCs in the SVZ and DG respond differently to various physiological and pathological events [12–14], we wondered whether lack of CPE affects hippocampal neurogenesis. Moreover, the role of CPE in adult hippocampal neurogenesis in the context of AD deserves exploration.

MicroRNA (miRNA) is a type of small non-coding RNA that fine-tunes gene expression post-transcriptionally. Most miRNAs are known to bind to the 3′-untranslated region (3′UTR) of target genes to downregulate their expression. However, certain miRNAs have been reported to upregulate the expression of native genes through targeting their 5′UTRs [15]. Recently, emerging miRNA therapeutics are offering a new promise in a range of health conditions, including neurodegeneration. This is attributed to their desirable features for drug development, such as inter-species conservation, short ~ 22 nucleotides in length facilitating drug design, and the potential for in vivo delivery via approved delivery system [16, 17]. Compared to miRNA mimics that are easily degraded, miRNA agomir, a chemically modified miRNA mimic, is more stable. Therefore, exploring miRNA agomirs targeting important disease-related factors will provide a great opportunity for disease treatment.

We herein sought to explore the role of CPE in adult hippocampal neurogenesis and AD-associated pathologies using aged and APP/PS1 mice, and to identify potential targeted therapeutic drugs. First, we detected the expression changes in CPE and BDNF-TrkB pathway in the hippocampus of four different ages spanning the lifespan and 9-month-old APP/PS1 mice, and found that the hippocampal level of CPE and the mBDNF/proBDNF ratio decreased significantly in the aged and APP/PS1 mice. Further, through lentivirus-mediated intervention to modulate CPE expression, we observed that hippocampal CPE deficiency led to impaired maturation of BDNF and significantly decreased adult neurogenesis, whereas exogenous supplementation of CPE promoted adult neurogenesis. These findings indicate that CPE may be a critical regulator of adult neurogenesis in the hippocampus. To develop effective therapeutic strategies, we designed and screened miRNA agomirs targeting the 5’ UTR of CPE to upregulate its expression. The effects of miRNA agomirs on adult neurogenesis, learning and cognitive abilities, and AD pathology in aged and APP/PS1 mice were assessed through intracerebroventricular (icv) injection or non-invasive intranasal instillation. Our results show that the miRNA therapy targeting CPE can significantly improve adult neurogenesis, rescue memory deficits and ameliorate AD-linked pathologies, thus offering a potentially effective therapeutic strategy for counteracting aging- and disease-related neurological decline in human brains.

## Methods

### Animals

Wild-type (WT) mice (C57BL/6J strain) were obtained from Beijing Vital River Laboratory Animal Technology Co., Ltd. (Beijing, China). Female APPswe/PSEN1ΔE9 double transgenic mice (referred to as APP/PS1 mice in the following) were obtained from Beijing HFK Bio-technology Co., Ltd., Institute of Laboratory Animal Science, Chinese Academy of Medical Science (Beijing, China). All animals were housed in temperature- and humidity-controlled rooms with ad libitum access to food and water.

Young (2–3 months old [MO]) and middle-aged (7–9 MO) WT mice were bred and aged in house for biochemical and immunostaining studies. Middle-aged WT mice were treated with lentivirus, miRNA mimics and agomirs via icv or intrahippocampal injection. One week later, the mice were anesthetized by an intraperitoneal injection of sodium pentobarbital (70 mg/kg) and perfused transcardially with ice-cold PBS followed by 4% paraformaldehyde (PFA), and brain tissues were collected for biochemical and immunostaining studies. The 9-MO female APP/PS1 mice were treated with miRNA agomirs by icv injection and intranasal instillation, and the brain samples were taken for neurogenesis experiments (2 weeks after icv injection, or on day 21 of intranasal instillation) and dendritic branching analyses (3 weeks after icv injection, or on day 21 of intranasal instillation). At the age of 10–11 MO, behavioral tests and AD-linked pathological analyses were performed.

### Cell lines

293T, MEF and N2a cells (ATCC, Rockville, MD) were cultured in Dulbecco’s Modified Eagle Medium supplemented with 10% fetal bovine serum at 37°C in 5% CO_2_. All the cell cultures were tested negative for mycoplasma contamination.

### Western blotting

Cell lysates and mouse brain tissues comprising the hippocampi were prepared as previously described [7]. The protein extractions were analyzed by standard western blotting procedures and bands were visualized by Chemilluminescence. The following primary antibodies were used: mouse anti-actin (1:50,000, Proteintech, 66009-1-Ig, Rosemont, IL), rabbit anti-BDNF (1:3000, Genetex, GTX134514, Irvine, CA), rabbit anti-proBDNF (1:1000, Thermo Fisher Scientific, PA5-77533, Carlsbad, CA), rabbit anti-TrkB (1:1000, Proteintech, 13129-1-AP), rabbit anti-CPE (1:1000, Proteintech, 13710-1-AP), and rabbit anti-FGF2 (1:1000, Bioss, bs-0217R, Beijing, China).

### In situ hybridization

In situ hybridization was carried out as previously described [7]. Digoxigenin-labeled CPE antisense oligonucleotide probe was synthesized by in vitro transcription using the NTP labeling mix from Roche (11277073910, Basel, Switzerland) and T7 RNA polymerase from Promega (P2077, Madison, WI).

### Immunostaining

Immunostaining for brain slices was performed as described previously [7], in which the primary antibodies were also listed. New antibodies used in this study include: thioflavin S (MCE, HY-D0972, Monmouth Junction, NJ), anti-C12orf34 (aggregatin) (1:100, Abcam, ab122626, Boston, MA), anti-Aβ (1:100, Proteintech, 60342-1-Ig), anti-synaptophysin (1:100, Proteintech, 17785-1-AP), anti-AT8 (1:100, Invitrogen, MN1020, Carlsbad, CA), anti-ApoE (1:100, Abcam, ab20874), anti-pTau-S396 (1:100, Thermo Fisher Scientific, 44-752G), and anti-Secretogranin III (1:100, Proteintech, 10954-1-AP).

### Quantitative real-time PCR (qRT-PCR)

Total RNA was extracted from the hippocampal tissues with TRIzol™ Reagent (Invitrogen) according to the manufacturer’s instructions, and was reverse-transcribed using the GoStript™ Reverse Transcription System (Promega). qRT-PCR was performed with the Hieff^®^ qPCR SYBR Green Master Mix (YEASEN, Shanghai, China). GAPDH was used as a normalization control. The primers are as follows: CPE forward 5’-CAGCAAGAGGACGGCATCTC-3’, reverse 5’-GTCCAACCGCCTCATTACCAT-3’; BDNF forward 5’-TCATACTTCGGTTGCATGAAGG-3’, reverse 5’-AGACCT-CTCGAACCTGCCC-3’; GAPDH forward 5’-AGGTCGGTGTGAACGGATTTG-3’, reverse 5’-TGTAGACCATGTAGTTGAGGTCA-3’.

### BrdU labelling experiments

For analysis of cell proliferation in the adult mouse brain, adult mice were given three injections of BrdU (Roche, 50 mg/kg body weight, i.p.) within 24 h, and the animals were sacrificed 24 h following the last BrdU injection by intraperitoneal injection of pentobarbital followed by transcardial perfusion with 4% PFA. Brains were collected and processed for routine OCT (optimal cutting temperature compound) embedding and sectioning. For quantification of BrdU-positive cells in the adult brain, free-floating sections were treated with 2N HCl at 37ºC for 30 min, and neutralized by washing with 0.1 M sodium borate buffer (pH 8.5) for 10 min at room temperature. Sections were blocked in 5% donkey serum in Tris-buffered saline plus 0.5% Triton X-100 for 1 h, and subsequently incubated with rat anti-BrdU antibody (1:500, Abcam, ab6326) overnight at 4ºC, followed by donkey anti-rat Alexa Fluor 488 nm antibody (1:1000, Invitrogen, A21208) for 2 h.

### Recombinant lentivirus production

For CPE overexpression, *CPE* cDNA was cloned into the modified SBP-Flag-tagged pWPXL lentiviral vector. The details of the construction are available upon request. Lentiviral particles were prepared by transiently transfecting 293T cells with lentiviral vectors together with packaging vectors pMD2.G and psPAX2 by using PEI as described previously [18]. The supernatants from three 10-cm dishes collected at 48 h and 72 h post-transfection were pooled and filtered through a 0.45-μm filter. Viruses were concentrated by ultracentrifugation at 100,000 g for 2 h at 4ºC. The virus particles were then washed once, and re-suspended in 20 µl PBS. We routinely obtained 1 × 10^9^ infectious lentiviral particles/ml.

### Isolation, culture and lentiviral infection of primary NSCs

Primary NSCs were isolated from the DG of 8-week-old WT mice based on published methods [19]. The NSCs were cultured in 24-well culture plates after the initial passage and concentrated lentiviruses of CPE and empty vector (as a control) were prepared as described above. For typical infection of primary NSCs, we added 1 μl of 1 × 10^9^/ml concentrated lentiviral particles to 500 μl of Neurobasal-A medium with B27-A in each well of the 24-well plate. The medium was replaced with fresh medium 12 h after infection. Western blotting was performed 48 h later.

### Stereotaxic injections

Lentivirus expressing CPE (LV-CPE) was injected stereotaxically into the DG in the right hemisphere, and a similar titer of lentivirus expressing the vector was injected into the left hemisphere as a control. The volume injected into each hippocampus was 2 µl, with a rate of 0.2 µl per minute. The injection coordinates were: 2.3 mm posterior relative to the bregma, ± 1.6 mm lateral to the midline, and 2.3 mm ventral to the surface of the skull.

For SVZ injection, 2.5 µl of virus particles were injected stereotaxically into one side of the SVZ. The coordinates for icv injection into the left lateral ventricle were: 0.5 mm anterior to the bregma, 1.3 mm lateral to the midline, and 2.9 mm ventral to the surface of the skull.

### Treatment of mice with the miRNA mimics or agomirs

The miRNA mimics were purchased from Genepharma (Shanghai, China). miRNA agomirs and negative control (NC) agomirs were obtained from RiboBio (Guangzhou, China). For icv/DG delivery, the NC, miRNA mimics (0.6 nmol) or miRNA agomirs (1 nmol) were injected into the lateral ventricles/DG using a microsyringe (Hamilton) under the guidance of a stereotaxic instrument (RWD Life Science, Shenzhen, China). The needle was left in place for an additional 5 min after injection to prevent possible leakage. Stereotaxic coordinates were the same as mentioned above. At 2 or 3 weeks post icv injection, 3–5 mice from each group were sacrificed, and brain tissue samples were taken for neurogenesis experiments (2 weeks) and dendritic branching analyses (3 weeks). At 4 weeks post icv injection, the mice were trained and tested on the Morris water maze (MWM). At 5 weeks post icv injection, AD-related pathological analyses were performed.

For intranasal instillation, 1 nmol agomirs were administered by applying a series of six 4-μl drops (a total of 24 μl) with a micropipette to the nasal cavity, alternating between the right and the left nostril with 1 min intervals. The instillation was performed every other day for a total of 30 days. On day 21, six mice from each group were sacrificed, and brain tissue samples were taken for neurogenesis and dendritic branching analyses. At 1 to 4 weeks after completion of the 30-day agomir treatment, MWM or Barnes maze was performed. At 5 weeks after completion of the 30-day agomir treatment, AD-related pathological analyses were performed.

### Microscopy, image acquisition and analysis

For immunolabelled mouse brain sections, images were acquired using a Nikon A1R Eclipse Ti confocal microscope (Nikon, Japan). For laser scanning confocal microscopy, the z-stacks spanning 25–30 μm, with serial optical sections of 1.5 μm were recorded. ImageJ was used for image processing and quantification. Stereology was performed as described [20] for adult neurogenesis analyses in the DG/SVZ region. The DG was analyzed comprising the region between 1.2 and 2.3 mm posterior to bregma, while the SVZ was between 1.18 mm anterior and 0.02 mm posterior to the bregma. BrdU^+^ and BrdU^+^doublecortin (DCX)^+^ cells within the DG/SVZ were counted using every 6^th^ section (180 μm apart), and the number of counted cells in the randomly selected section was normalized to the area of the DG, then multiplied by six. The total positive cell number in the entire DG/SVZ region was calculated by accumulating the counts across all sections.

ImageJ was used to measure the mean fluorescence intensity. The protein fluorescence levels in the subgranular zone (SGZ, defined as a 2-cell-body-wide zone along the border of the granule cell layer) were determined by the mean intensity, and were normalized to the DG area of each section. At least three brain sections at different rostral/caudal levels from each animal were randomly selected, analyzed and averaged, and at least three animals in each group were used for analysis.

For dendritic branching analyses, Z-stack images were captured from slides using identical settings for Sholl analysis and total dendritic length measurement of each neuron. In each group, 20–25 neurons from 3 animals were analyzed. Images were processed using the ImageJ software.

For analysis of the perikaryon area of GFAP^+^ astrocytes, ImageJ was used to outline the cytoplasmic region of astrocytes and measure the area. At least 30 astrocytes were counted per brain section, and with three sections per mouse for the mean. For the percent area of GFAP^+^ astrocytes in the hippocampus, ImageJ was used to measure the whole cell area of astrocytes and calculate the percentage relative to the selected area of the hippocampus. Three different areas were selected per section and three sections per mouse for the mean. At least three animals in each group were used for analysis.

### MWM test

The spatial learning and memory of mice was tested using the MWM test, and memory flexibility and the ability to adjust innate cognition were tested with spatial reversal of the platform in the MWM test. MWM test was performed as described with modifications [21]. Briefly, mice were trained in a round white pool with a diameter of 120 cm that was filled with opaque water at approximately 22 ± 1ºC. An escape platform (10 cm in diameter) was placed in one of the quadrants 1 cm beneath the water surface. Maze cues with different shapes, colors and sizes were posted on the wall surrounding the water tank in four directions. Mice were trained for 5 days and then subjected to four trials per day, each with a different start position in different quadrants. If a mouse failed to find the hidden platform within 60 s, it was manually guided to the hidden platform and allowed to rest on it for 15 s. The 5-day basic acquisition training mainly recorded the latency to find the platform to draw a learning curve. Twenty-four hours after the last acquisition day, a 60-s spatial probe trial was performed. For the probe trial, the platform was removed and the mouse was placed in the maze at a position farthest from the original platform area. For spatial reversal of MWM, the hidden platform was switched to the diagonal quadrant, and the starting position for each trial during the acquisition phase was adjusted accordingly. The remaining procedures were performed as above. The latency, the staying time, the number of crossings in the original location of the platform and the swimming velocity were automatically monitored with a video tracking system (SMART Video Tracking System). All data were analyzed using the SMART v3.0 software (Panlab, Harvard Apparatus, Holliston, MA). Nine to 11 mice per group were analyzed and assessed for statistical significance.

### Barnes maze

Mice were also analyzed for neurocognitive deficits and spatial navigation using Barnes maze. The Barnes maze consisted of a white circular acrylic platform, 92 cm in diameter and 82 cm above the floor, with 20 equally spaced holes (5 cm diameter) around the perimeter. One of the holes is the target escape that led to a box beneath the surface. For aversive stimulations, intense light was used. A tracking camera device connected to a computer was positioned over the maze to capture behavioral experiments. Multi‐colored visual cues were placed around the room in the four cardinal directions, north, south, east and west. The animal was released in the center of the maze with the lights dimmed. The bright lights overhead were turned on, and the mouse was given 5 min to explore the maze. If it did not enter the escape box, it was guided to the escape hole and left there for 30 s before being returned to its home cage. The learning phase (day 1 to day 5) consisted of 2 × 3-min trials per day with an inter-trial interval of 20–25 min. After each trial, the maze’s surface was wiped clean with 70% ethanol and dried. For the probe trial on day 6, the escape box was replaced with a standard hole and the mice were tested for 90 s. The latency was recorded. Also, target poke on probe trial (visit to target) was measured. Seven to 14 mice per group were analyzed and assessed for statistical significance.

### Statistical analysis

For all experiments, at least three biological or experimental replicates were analyzed. Sample size for each experiment is indicated in the corresponding figure legend. All data are presented as mean ± SEM unless stated otherwise. Statistical significance of data from two groups was analyzed using two-tailed unpaired Student’s *t* test. Data from multiple groups were analyzed with one-way analysis of variance (ANOVA) followed by Dunnett’s multiple comparisons test. *N* represents the number of evaluated animals. Significance was set at *P* < 0.05.

## Results

### The age-related decline of CPE causes deficiency of the BDNF–TrkB signaling in the hippocampus

**Fig. 1:**
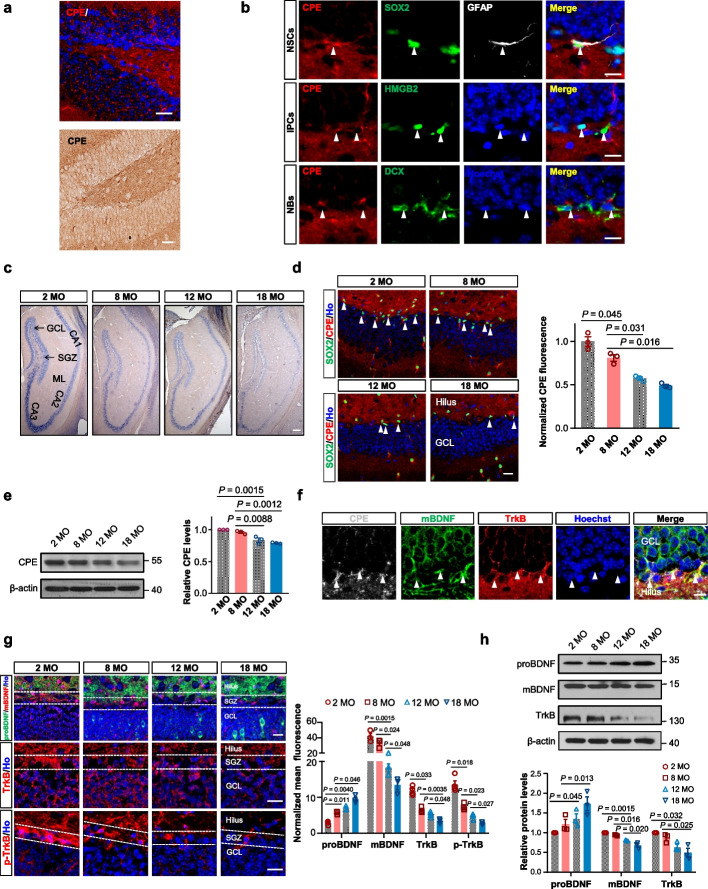
CPE and the BDNF–TrkB signaling in the hippocampus are downregulated during aging. **a** The CPE protein expression in the DG of 2-MO WT mice. Ho, Hoechst. Scale bars, 20 μm. **b** Characterization of CPE-positive NSCs in the SGZ of 2-MO WT mice. White arrowheads indicate SOX2^+^CPE^+^GFAP^+^ NSCs (top panels), CPE^+^HMGB2^+^ intermediate progenitor cells (IPCs, middle panels) and CPE^+^DCX^+^ neuroblasts (NBs, bottom panels) in the SGZ. Scale bars, 10 µm. **c** In situ hybridization analysis of CPE mRNA in the hippocampus of WT mice at 2, 8, 12 and 18 MO. Scale bar, 100 µm. GCL, granular cell layer; SGZ, subgranular zone; ML, molecular layer. **d** Representative images and normalized fluorescence intensity of CPE expression in the SGZ of WT mice at 2, 8, 12 and 18 MO. Scale bar, 20 µm. White arrowheads indicate SOX2^+^CPE^+^ cells in the SGZ. **e** Western blotting analyses of proteins extracted from the mixed tissues of the hippocampus from three WT mice at each age. Relative quantification of CPE levels in the DG is shown on the right. **f** Representative images of SGZ immunostaining for CPE, mBDNF and TrkB of 2-MO WT mice. Scale bar, 10 μm. White arrowheads indicate TrkB^+^CPE^+^mBDNF^+^ cells. **g** Representative images and normalized fluorescence intensity of proBDNF/mBDNF, TrkB and p-TrkB expression in the DG of WT mice at 2, 8, 12 and 18 MO. Scale bars, 10 µm. Dashed lines indicate the SGZ. **h** Western blotting analyses of proteins extracted from the mixed tissues of the hippocampus from three WT mice at each age (upper panel). Relative quantification of each protein level in the DG is shown in the lower panel. For (**d**), (**e**), (**g**) and (**h**), data are presented as mean ± SEM. *n* = three/four mice each age. Data were analyzed with one-way ANOVA

To determine whether CPE is involved in adult hippocampal neurogenesis, we first assessed whether CPE is expressed in the DG. CPE expression was detected in most of the granule neurons in the DG (Fig. 1a). In the SGZ, CPE was expressed in SOX2^+^GFAP^+^ NSCs, HMGB2^+^ proliferating neuronal precursor cells and DCX^+^ immature neurons (Fig. 1b). In the hippocampus, intense labelling of *Cpe* mRNA was observed throughout the pyramidal cell layer of CA1 and CA3 and in the granule cell layer of DG (Fig. 1c). We then determined the time course of CPE expression with age, and found that the signals of mRNA transcripts (Fig. 1c) as well as protein levels (Fig. 1d, e) of CPE in the hippocampus declined progressively with age.

Recently, we have discovered a coordinated regulation between CPE and mature BDNF (mBDNF) production in the SVZ [7]. Here, we investigated whether it is also the case in the DG. Similarly, TrkB, p-TrkB and mBDNF were expressed in NSCs and their progeny in the SGZ (Additional file 1: Fig. S1a–c). Triple immunostaining confirmed the co-localization of CPE, mBDNF and TrkB in the SGZ (Fig. 1f). Furthermore, the levels of mBDNF, TrkB and p-TrkB gradually declined with age (Fig. 1g, h), indicating that a reduction in CPE in the hippocampus may also contribute to the defects in the mBDNF–TrkB signaling in aged brains.

### CPE regulates the hippocampal neurogenesis via the BDNF–TrkB signaling pathway

**Fig. 2:**
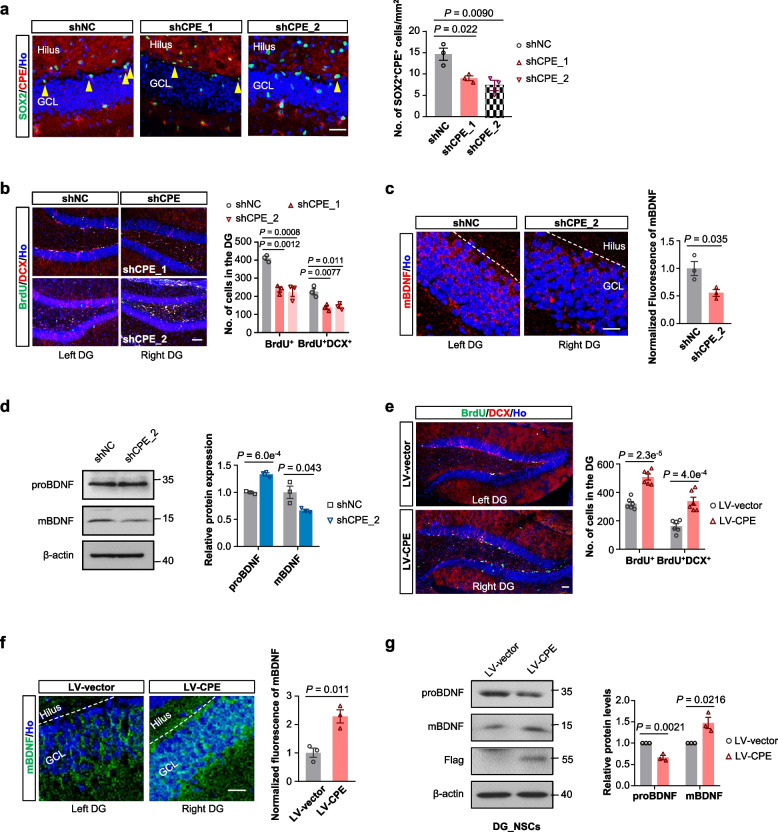
CPE promotes adult neurogenesis and BDNF generation in the hippocampus in vivo. **a** Representative images and quantification of SOX2^+^CPE^+^ cells in the DG of middle-aged WT mice 1 week after LV-shNC, shCPE_1 or shCPE_2 injection. Scale bar, 10 µm. Data are presented as mean ± SEM, *n* = three mice each group. One-way ANOVA. **b** Representative images and quantification of BrdU and DCX double-labelled newly generated neurons in the DG of the same brain from middle-aged WT mice 1 week after LV-shNC (left DG), shCPE_1 or shCPE_2 (right DG) injection. Scale bar, 20 µm. Data are presented as mean ± SEM, *n* = three mice each group. One-way ANOVA. **c** Representative images and normalized fluorescence intensity of mBDNF expression in the DG of middle-aged WT mice 1 week after LV-shNC (left DG) or shCPE_2 (right DG) injection. Scale bar, 10 µm. Data are presented as mean ± SEM, *n* = three mice each group. **d** Western blotting analyses and relative quantification of proteins extracted from the mixed tissues of hippocampus from three middle-aged WT mice 1 week after LV-shNC or shCPE_2 injection. Data are presented as mean ± SEM from three mice each group. **e** Representative images and quantification of BrdU and DCX double-labelled newly generated neurons in the DG of the same brain from middle-aged WT mice 1 week after LV-vector (left DG) or LV-CPE (right DG) injection. Scale bar, 20 µm. Data are presented as mean ± SEM, *n* = six mice each group. **f** Representative images and normalized fluorescence intensity of mBDNF expression in the DG of middle-aged WT mice 1 week after LV-vector (left DG) or LV-CPE (right DG) injection. Scale bar, 20 µm. Data are presented as mean ± SEM, *n* = three mice each group. **g** Western blotting analyses and relative quantification of proteins extracted from cultured primary NSCs isolated from hippocampus and infected with lentivirus vector or lentivirus expressing CPE. Data are presented as mean ± SEM from three independent experiments. For (**c**–**g**), data were analyzed with two-tailed Student’s *t*-test

To investigate whether CPE plays a role in adult hippocampal neurogenesis and if the BDNF–TrkB signaling is involved in this process, we injected lentivirus expressing shRNA targeting CPE or negative control shRNA (shNC) in the right DG and the left DG of mice, respectively. Two different shRNAs against the mouse *Cpe* gene were designed (Additional file 1: Fig. S2a). One week after lentivirus injection, BrdU and DCX immunohistochemistry was used to quantify neurogenesis in the DG. Effective and sustained reduction of CPE was observed after a single injection (Fig. 2a). Likewise, the decrease of CPE led to significant reduction of hippocampal neurogenesis (Fig. 2b) and mBDNF levels (Fig. 2c, d) compared to LV-shNC controls. In addition, no significant difference was observed in the TUNEL-positive cell counts in the SGZ between the CPE knockdown and negative control groups (Additional file 1: Fig. S2b), thus excluding the involvement of apoptosis in this process.

We also stereotaxically injected lentiviruses expressing vector (LV-vector) or CPE (LV-CPE) in the left and the right hippocampus, respectively. Injection of LV-CPE resulted in significant increases in CPE expression (Additional file 1: Fig. S2c), numbers of BrdU^+^ and BrdU^+^DCX^+^ cells (Fig. 2e), and mBDNF immunofluorescence staining (Fig. 2f) in the DG of the ipsilateral compared to the LV-vector side. These results demonstrate that CPE can promote adult hippocampal neurogenesis in vivo. In support of this, similar increases were observed when CPE protein was intracerebroventricularly infused in mice (Additional file 1: Fig. S2d–f), which could be abolished by ANA-12, a highly potent and selective TrkB receptor antagonist (Additional file 1: Fig. S2g). In line with these in vivo results, increased mBDNF levels were observed when CPE was overexpressed in cultured primary NSCs derived from hippocampi (Fig. 2g), indicating that CPE regulates the BDNF–TrkB signaling directly within the NSC lineages in the hippocampus. Taken together, these data support a crucial role of CPE in adult hippocampal neurogenesis and BDNF–TrkB signaling regulation in NSCs of DG.

### Increasing CPE via miRNA agomirs restores hippocampal neurogenesis

**Fig. 3:**
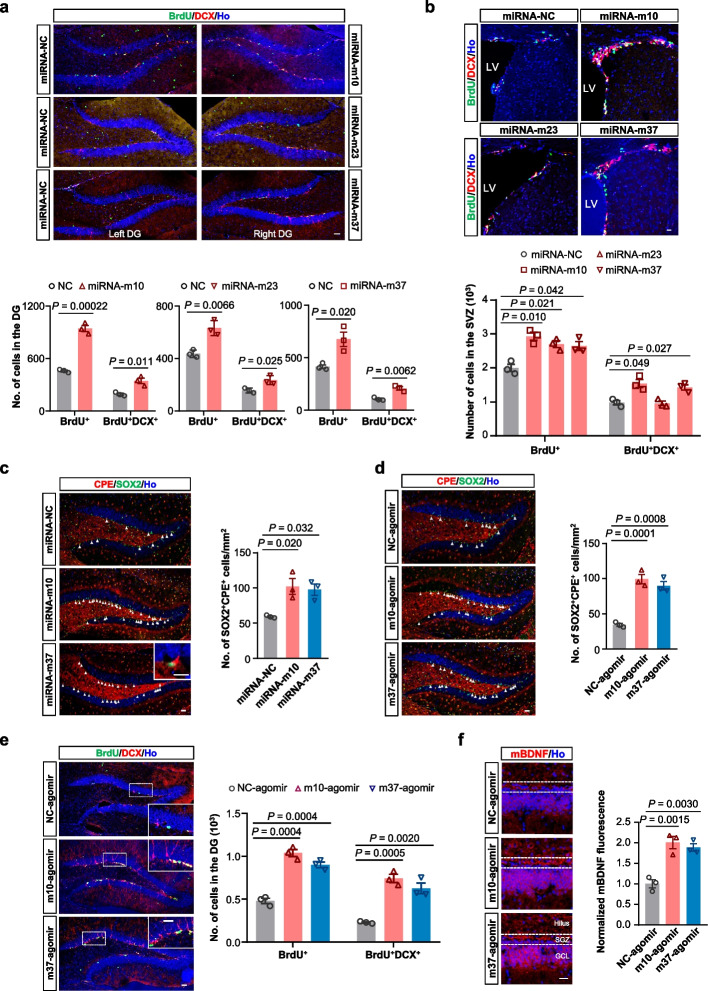
CPE-upregulating miRNA agomirs promote hippocampal neurogenesis and mBDNF generation in middle-aged WT mice. **a**, **b** Representative images (upper panel) of BrdU and DCX double-labelled newly generated neurons in the DG (**a**) or SVZ (**b**) of middle-aged WT mice 1 week after miRNA mimic icv injection. Scale bars, 20 µm. Quantifications of the number of BrdU^+^ and BrdU^+^DCX^+^ cells are shown in the lower panel. Data are presented as mean ± SEM, *n* = three mice each group. Two-tailed Student’s *t*-test. **c**, **d** Representative images (left) and quantification (right) of SOX2^+^CPE^+^ cells in the SGZ of middle-aged WT mice 1 week after icv injection of NC-, m10- or m37-miRNA mimics (**c**) or agomirs (**d**). The inset in (**c**) shows magnified image of SOX2^+^CPE^+^ cells in the SGZ. Ho, Hoechst. Scale bars, 50 µm. Data are presented as mean ± SEM, *n* = three mice each group. **e** Representative images of BrdU and DCX double-labelled newly generated neurons in the DG of the brain from middle-aged WT mice 1 week after miRNA agomir icv injection. Scale bars, 20 µm. The white boxes represent the area imaged with a higher magnification. Quantification of BrdU^+^ and BrdU^+^DCX^+^ cells in the DG is shown on the right. Data are presented as mean ± SEM, *n* = three mice each group. **f** Representative images of mBDNF expression in the DG of middle-aged WT mice 1 week after miRNA agomir icv injection. Ho, Hoechst. Scale bar, 20 µm. Normalized mBDNF fluorescence intensity in the DG is shown on the right. Data are presented as mean ± SEM, *n* = three mice each group. For (**b**–**f**), data were analyzed with one-way ANOVA

Next, we screened 44 synthetic miRNA mimics predicted to bind the 5’UTR of *Cpe* using miRWalk (Table 1), looking for candidates that could upregulate CPE expression in N2a cells. Among them, eight miRNA mimics significantly increased the CPE level at 30 h post-transfection, and were selected to evaluate their promoting effects on adult hippocampal neurogenesis in mice (Additional file 1: Fig. S3a). Among them, three mimics, m10, m23, and m37, enhanced DG neurogenesis in the middle-aged WT mice (Fig. 3a). Moreover, m10 and m37, but not m23, also significantly enhanced adult SVZ neurogenesis when stereotactically injected into the mouse SVZ (Fig. 3b). Furthermore, an upregulation of CPE protein was detected in the DG 1 week after the m10 and m37 miRNA injection (Fig. 3c), confirming that these miRNAs indeed upregulated endogenous CPE expression in vivo. Consistently, miRNA agomirs of m10 and m37 (m10-agomir and m37-agomir), which are chemically modified stable miRNA mimics, also increased CPE expression (Fig. 3d) and hippocampal neurogenesis (Fig. 3e) after injection in mice. Meanwhile, both m10- and m37-agomir injections resulted in higher levels of mBDNF (Fig. 3f).

**Table 1:** List of microRNAs predicted by miRWalk to bind with the 5’UTR of *Cpe* gene

**Number**	**MicroRNA name**	**Sequence**	
		**Sense (5’-3’)**	**Antisense (5’-3’)**
m1	mmu-miR-7060-3p	UCUACUCUACCUUCUACUCAG	GAGUAGAAGGUAGAGUAGAUU
m2	mmu-miR-702-3p	UGCCCACCCUUUACCCCGCUCC	AGCGGGGUAAAGGGUGGGCAUU
m3	mmu-miR-7088-3p	UUGACCUUCCUCCAUUGCUUCC	AAGCAAUGGAGGAAGGUCAAUU
m4	mmu-miR-6896-3p	UUUCUCUCUCUCACCUACAAAC	UUGUAGGUGAGAGAGAGAAAUU
m5	mmu-miR-3059-5p	UUUCCUCUCUGCCCCAUAGGGU	CCUAUGGGGCAGAGAGGAAAUU
m6	mmu-miR-6917-3p	GUCACUUCUCUUCCCACCACAG	GUGGUGGGAAGAGAAGUGACUU
m7	mmu-miR-6963-3p	UGCCUCUUGCCUCCAUCCCACAG	GUGGGAUGGAGGCAAGAGGCAUU
m8	mmu-miR-6975-3p	UCUCUCCUUUCUCCUCCUAG	AGGAGGAGAAAGGAGAGAUU
m9	mmu-miR-7009-3p	UCUUUUCCCCUCUCCCUGCAG	GCAGGGAGAGGGGAAAAGAUU
m10	mmu-miR-7031-3p	AACCCUCUUGCCCUCUCCUAG	AGGAGAGGGCAAGAGGGUUUU
m11	mmu-miR-7032-3p	AUCCUCUCGGUACCGCCCUGCA	CAGGGCGGUACCGAGAGGAUUU
m12	mmu-miR-7650-5p	AAUCCUCUUGCAACCCAGAACU	UUCUGGGUUGCAAGAGGAUUUU
m13	mmu-miR-8112	UCUCCGCCACCUCCACCGCA	CGGUGGAGGUGGCGGAGAUU
m14	mmu-miR-1224-3p	CCCCACCUCUUCUCUCCUCAG	GAGGAGAGAAGAGGUGGGGUU
m15	mmu-miR-3099-5p	CCCCACCUCUUCUCUCCUCAG	GAGGAGAGAAGAGGUGGGGUU
m16	mmu-miR-6978-3p	ACGGCUUCACUCUCACCCUGCAG	GCAGGGUGAGAGUGAAGCCGUUU
m17	mmu-miR-7068-5p	GUGAGGCUCAGUAUGGGGUGG	ACCCCAUACUGAGCCUCACUU
m18	mmu-miR-7077-3p	CCUUCCAUGGCUCCUGGCAG	GCCAGGAGCCAUGGAAGGUU
m19	mmu-miR-7091-3p	AGUGGCUUCUGUCGUCUCUAG	AGAGACGACAGAAGCCACUUU
m20	mmu-miR-7220-5p	GGUGAGCUCUUGGUACCUUGGC	CAAGGUACCAAGAGCUCACCUU
m21	mmu-miR-7667-5p	GAGCCAUCUCUCUAGCCCCUGA	AGGGGCUAGAGAGAUGGCUCUU
m22	mmu-miR-7676-3p	UCCGGUGCUCACUCUGCCCACA	UGGGCAGAGUGAGCACCGGAUU
m23	mmu-miR-7012-3p	UGACCUGUGGCUCCUCUCCCAG	GGGAGAGGAGCCACAGGUCAUU
m24	mmu-miR-7022-3p	ACAAGCCUGACCUCUGCCCCCA	GGGGCAGAGGUCAGGCUUGUUU
m25	mmu-miR-7075-3p	CAACCAUGUCUUCUUUCCCAG	GGGAAAGAAGACAUGGUUGUU
m26	mmu-miR-7117-5p	UCUGGGGGCUCAGCUGAGGAUA	UCCUCAGCUGAGCCCCCAGAUU
m27	mmu-miR-185-3p	AGGGGCUGGCUUUCCUCUGGU	CAGAGGAAAGCCAGCCCCUUU
m28	mmu-miR-302c-5p	GCUUUAACAUGGGGUUACCUGC	AGGUAACCCCAUGUUAAAGCUU
m29	mmu-miR-1947-3p	GCACUGAGCUAGCUCUCCCUCC	AGGGAGAGCUAGCUCAGUGCUU
m30	mmu-miR-3076-3p	CGCACUCUGGUCUUCCCUUGCAG	GCAAGGGAAGACCAGAGUGCGUU
m31	mmu-miR-6990-3p	AGCCCUGCCUCUUCCUGGCAG	GCCAGGAAGAGGCAGGGCUUU
m32	mmu-miR-7005-5p	CCUGGGGAUGGGAGGACCAGCA	CUGGUCCUCCCAUCCCCAGGUU
m33	mmu-miR-7684-3p	UGCUGACUGGGGCUGGCCUGUG	CAGGCCAGCCCCAGUCAGCAUU
m34	mmu-miR-7686-3p	CUGCUCGGGGCACUGUAAGAGA	UCUUACAGUGCCCCGAGCAGUU
m35	mmu-miR-338-3p	UCCAGCAUCAGUGAUUUUGUUG	ACAAAAUCACUGAUGCUGGAUU
m36	mmu-miR-106a-3p	ACUGCAGUGCCAGCACUUCUUAC	AAGAAGUGCUGGCACUGCAGUUU
m37	mmu-miR-1982-5p	UUGGGAGGGUCCUGGGGAGG	UCCCCAGGACCCUCCCAAUU
m38	mmu-miR-3090-5p	GUCUGGGUGGGGCCUGAGAUC	UCUCAGGCCCCACCCAGACUU
m39	mmu-miR-6340	GUCAGCAGCAGCUUCGCUUUGGC	CAAAGCGAAGCUGCUGCUGACUU
m40	mmu-miR-6366	AGCUAAGGGGCCCGGGGAGCCA	GCUCCCCGGGCCCCUUAGCUUU
m41	mmu-miR-12182-5p	ACAGCGCCAGCUGCCUAAUUGA	AAUUAGGCAGCUGGCGCUGUUU
m42	mmu-miR-6982-5p	CUGGAGGAUCGCAGGGGUGGCCUGG	AGGCCACCCCUGCGAUCCUCCAGUU
m43	mmu-miR-6900-3p	UGGUGAUGGGCUCUCUUGUAG	ACAAGAGAGCCCAUCACCAUU
m44	mmu-miR-713	UGCACUGAAGGCACACAGC	UGUGUGCCUUCAGUGCAUU

The number, name and sequence of 44 microRNAs predicted by miRWalk to bind with the 5’UTR of *Cpe* gene are listed

### miRNA agomirs restore hippocampal neurogenesis and promote dendrite development in APP/PS1 mice

**Fig. 4:**
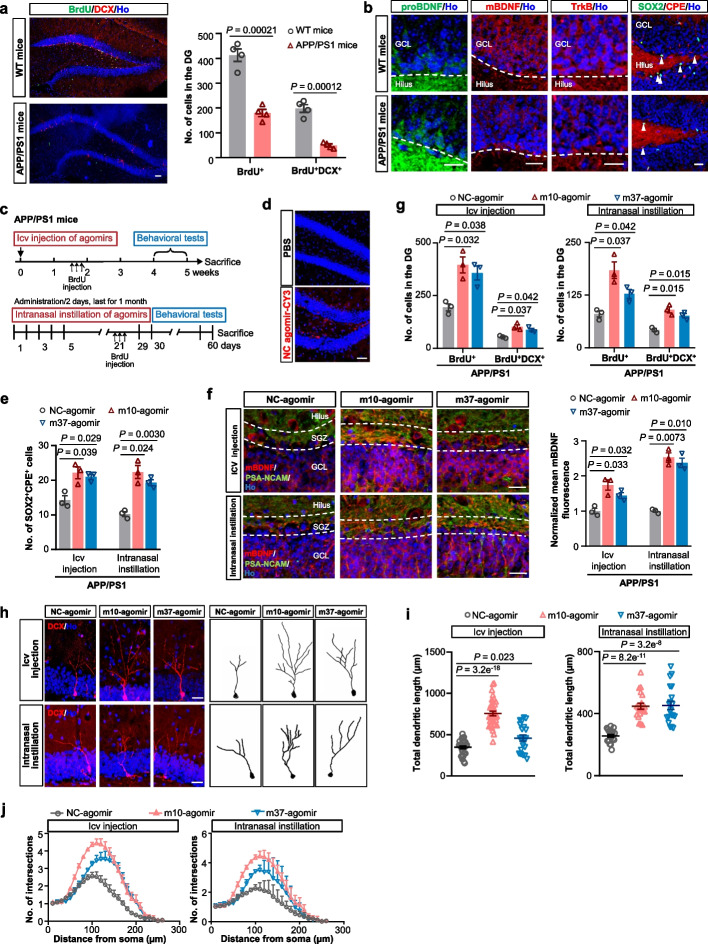
CPE-upregulating miRNA agomirs promote hippocampal neurogenesis, newborn-neuron dendrite development and mBDNF generation in APP/PS1 mice. **a** Representative images and quantification of BrdU and DCX double-labelled newly generated neurons in the DG of 9-MO WT and APP/PS1 mice. Scale bar, 100 µm. Data are presented as mean ± SEM, *n* = four mice each group. Two-tailed Student’s *t* test. **b** Representative images of proBDNF, mBDNF, TrkB and CPE expression in the DG of 9-MO female APP/PS1 transgenic mice compared with age-matched WT mice. Scale bars, 20 µm. Dashed line indicates the SGZ. Arrowheads indicate SOX2^+^CPE^+^ cells in the SGZ. **c** The timeline of the experiments for immunofluorescence and behavioral tests after intracerebreventricular (icv) injection or intranasal instillation of miRNA agomirs in APP/PS1 mice. **d** Hippocampi images of PBS or CY3-labeled NC-agomir (red) intranasally  delivered in 9-MO APP/PS1 mice. **e** Quantification of SOX2^+^CPE^+^ cells in the SGZ of 9-MO APP/PS1 mice 2 weeks after icv injection or on day 21 of intranasal instillation of miRNA agomirs. **f** Representative images and normalized fluorescence intensity of mBDNF and PSA-NCAM expression in the DG of 9-MO APP/PS1 mice treated with miRNA agomirs 2 weeks after icv injection or on day 21 of intranasal instillation. Scale bars, 20 µm. **g** Quantification of BrdU^+^ and BrdU^+^DCX^+^ cells in the DG of 9-MO APP/PS1 mice treated with miRNA agomirs 2 weeks after icv injection or on day 21 of intranasal instillation. **h** Representative images (Left) and sample traces (Right) of the morphology of DCX-positive immature neurons in the DG of 9-MO APP/PS1 mice 3 weeks following icv or intranasal delivery of agomirs. Scale bars, 20 µm. **i**, **j** Quantification of the total dendritic length (**i**) and dendritic complexity (**j**) of DCX-positive immature neurons in (**h**). *n* = 20–35 neurons from three mice each group. Data are presented as mean ± SEM. One-way ANOVA. For (**e**), (**f**) and (**g**), data are presented as mean ± SEM, *n* = three mice each group. Data were analyzed with one-way ANOVA

We then explored the therapeutic potential of m10 and m37 by evaluating their effects on adult hippocampal neurogenesis in APP/PS1 transgenic mice, a well-validated AD model [22]. Consistent with previous reports [23], APP/PS1 mice exhibited a remarkable decrease in hippocampal neurogenesis compared to age-matched WT controls (Fig. 4a). We detected a significantly decreased level of mBDNF, TrkB and CPE but a relatively higher level of proBDNF in the DG of 9-MO APP/PS1 mice compared to the age-matched WT mice (Fig. 4b and Additional file 1: Fig. S3b), suggesting the presence of an imbalance between pro-BDNF and mBDNF in AD mice. Therefore, the decreases of mBDNF and CPE in the hippocampus of APP/PS1 mice parallel the decreased proliferation of neuronal precursors in the DG.

Agomirs were administered to the APP/PS1 mice intracerebroventricularly. To test a noninvasive drug delivery route to the brain, the agomirs were also administered by intranasal instillation (Fig. 4c). Preliminary experiment using a Cy3-labeled NC-agomir showed that agomirs could be intranasally delivered to the hippocampi, demonstrating the feasibility of this noninvasive approach (Fig. 4d). When administered by icv injection or by intranasal instillation to the 9-MO APP/PS1 mice, both m10- and m37-agomirs increased CPE expression compared to the NC-agomir (Fig. 4e), accompanied by increases in mBDNF expression (Fig. 4f) and adult neurogenesis (Fig. 4g) in the DG. The CPE increase persisted at least until 11 MO (Additional file 1: Fig. S3c). Additionally, we found that the upregulated CPE induced by agomirs did not influence the transcriptional levels of BDNF (Additional file 1: Fig. S4a). We further observed changes of the mBDNF/proBDNF ratio in the same brain section after injection of m10-agomir or m37-agomir into the left lateral ventricle of APP/PS1 mice. The ipsilateral SVZ showed increased mBDNF and decreased proBDNF expression as compared to the contralateral side (Additional file 1: Fig. S4b). These results suggest that CPE contributes to the upregulation of mBDNF, acting at the post-transcriptional level. Moreover, consistent with previous reports [6, 24], the CPE increase caused by miRNA agomirs resulted in the upregulation of hippocampal FGF2 expression (Additional file 1: Fig. S4c, d), and the upregulated FGF2 may also play a crucial role in neurogenesis in APP/PS1 mice. Collectively, these results demonstrate that the CPE-targeting m10- and m37-agomirs are able to promote hippocampal neurogenesis in APP/PS1 mice, possibly by upregulating the BDNF–TrkB signaling pathway and the FGF2-related pathway.

Intriguingly, we also observed that 3 weeks after m10-/m37-agomir administration either by icv injection or by intranasal instillation, newborn neurons in the DG of APP/PS1 mice exhibited longer dendrites and more dendritic complexity compared with those in NC-agomir-treated APP/PS1 mice (Fig. 4h–j), showing that the agomir treatments also significantly enhanced dendritic development.

### miRNA agomirs alleviate memory deficits in APP/PS1 mice

To investigate whether m10- or m37-agomir treatment has a functional behavioral consequence, we monitored the hippocampal-dependent spatial learning and memory of APP/PS1 mice by MWM test and Barnes maze test. The APP/PS1 mice were treated with agomirs either by direct icv injection or by intranasal instillation. For mice administered with icv agomirs, only MWM test for spatial acquisition was performed.

**Fig. 5:**
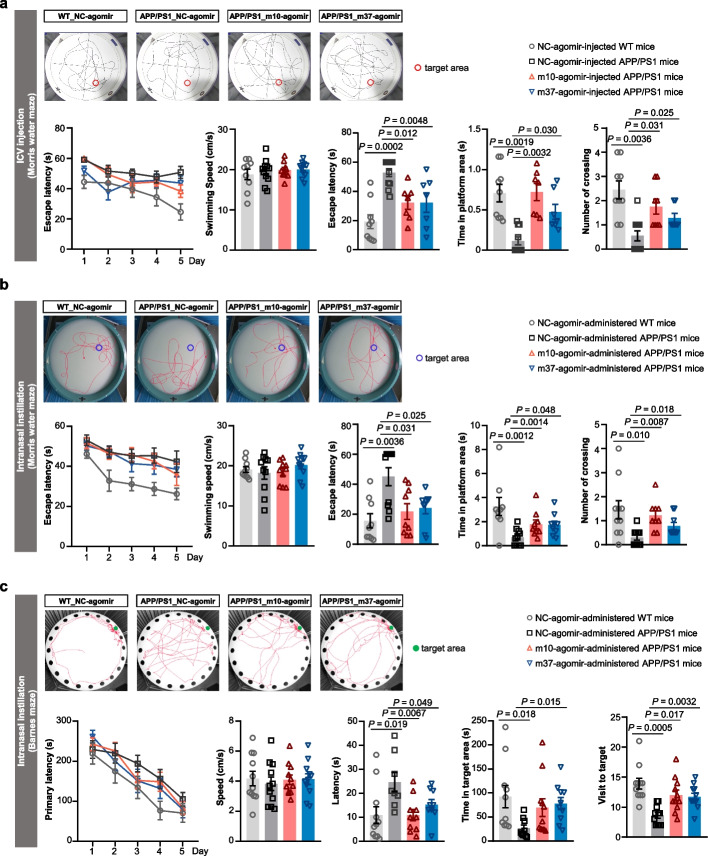
miRNA agomirs rescue memory deficits in APP/PS1 mice. **a**, **b** Rescue of behavioral deficits of 10–11-MO APP/PS1 mice treated with m10- or m37-agomir 4 weeks after icv injection (**a**) or 2 weeks after completion of the 30-day intranasal treatment (**b**) in the spatial acquisition of MWM test. Latency to reach the platform in the acquisition phase, swimming speed, escape latency to reach the original platform location, time in platform area and number of crossings during the probe trial are presented. Data are presented as mean ± SEM, *n* = 7–11 mice each group. **c** Rescue of behavioral deficit of 10-MO APP/PS1 mice treated with m10- or m37-agomir 1 week after completion of the 30-day intranasal treatment in Barnes maze. Latency, speed, time in the target area and target hole during the learning phase and the probe trial are presented. Data are presented as mean ± SEM, *n* = 11 mice each group. For (**a**–**c**), data were analyzed with one-way ANOVA

The APP/PS1 mice showed poor performance in both MWM and Barnes maze tests compared to age-matched WT mice after NC-agomir administration (Fig. 5a–c and Additional file 1: Fig. S4e). In contrast, m10- or m37-agomir treatment either by icv or by the intranasal route significantly improved the neurocognitive functions and memory flexibility in the MWM test, as indicated by the shorter escape latency and longer time in the platform quadrant compared to the NC-agomir group (Fig. 5a, b and Additional file 1: Fig. S4e). There was no difference in the swimming speed compared with the WT-NC-agomir group. In addition, APP/PS1 mice with intranasal m37-agomir treatment showed the same pattern of cognitive improvement in the Barnes maze, exhibiting significantly more visits of the target hole and longer time in the target area compared to the NC-agomir group (Fig. 5c). Treatment with m10-agomir also improved spatial memory, reflected by the significantly more visits of target hole but a non-significant trend of increased time in the target area (Fig. 5c). Together, our behavioral results indicate that both agomirs can reduce the deficits in spatial memory of APP/PS1 mice.

### miRNA agomirs ameliorate Aβ pathologies in APP/PS1 mice

**Fig. 6:**
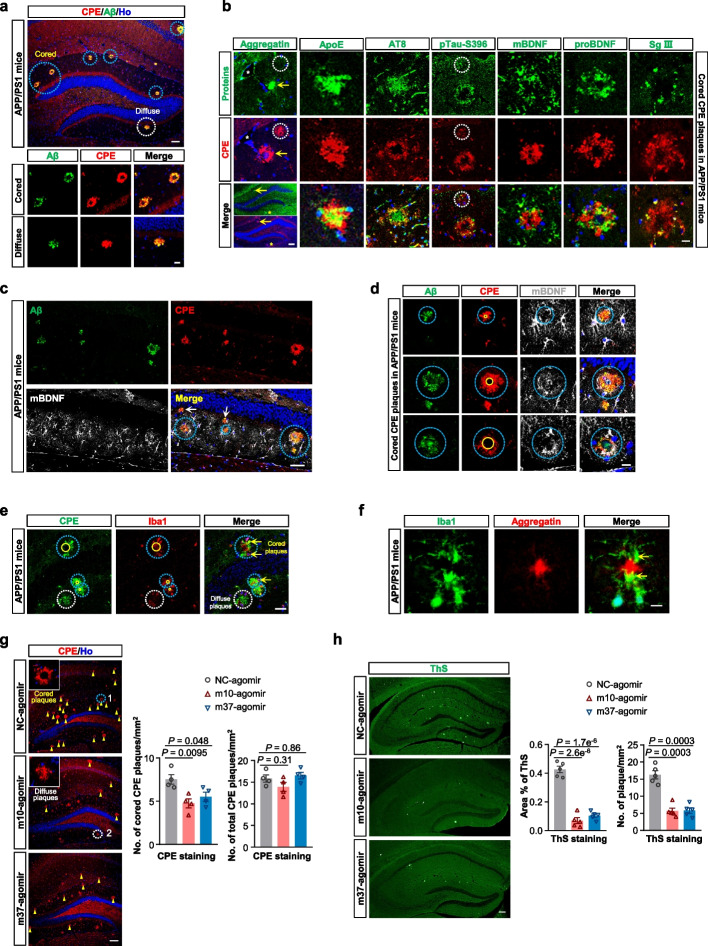
miRNA agomir treatment counteracts Aβ pathology in APP/PS1 mice. **a** The co-localization of Aβ plaques (stained by 6E10) and CPE in the hippocampus of 10–11-MO APP/PS1 mice. Blue dotted circles represent cored plaques while the white dotted circle indicates diffuse plaque. Scale bars, 100 μm (top panel), 20 μm (bottom panels). High magnifications of the two forms of Aβ plaques (cored and diffuse) are shown in the lower panels. **b** Various protein immunoreactivity of the cored CPE plaques in the hippocampus of 10–11-MO APP/PS1 mice. The leftmost panels show immunostaining for cored CPE plaque and aggregatin (yellow arrows) in adjacent sections (indicated by features marked with asterisks) of hippocampus in APP/PS1 mice. The white dotted circles indicate diffuse plaque. Scale bars, 100 μm (bottom left panel), 20 μm (other panels). **c** mBDNF immunoreactivity of the cored and diffuse CPE plaques in the hippocampus of 10–11-MO APP/PS1 mice. Scale bar, 20 μm. **d** Aβ and mBDNF immunoreactivity for the cored CPE plaques in the hippocampus of 10–11-MO APP/PS1 mice. Yellow circles indicate the growing of the CPE plaque core. Scale bar, 20 μm. **e** CPE plaques in the hippocampus of 10–11-MO APP/PS1 mice were associated with clusters of activated microglia (Iba1-positive). Scale bar, 20 μm. **f** Iba1-positive microglial cell bodies were located in close vicinity to the aggregatin-positive plaque core. Yellow arrows indicate microglial cell bodies. Scale bar, 10 μm. **g** Representative images and quantification showed that cored but not total CPE plaques (stained by CPE, indicated by yellow triangles) were reduced in the DG of 10–11-MO APP/PS1 mice 5 weeks after the completion of agomir treatment. Scale bar, 100 μm. Data are presented as mean ± SEM, *n* = four mice each group. **h** Fibrillar Aβ plaques (stained by ThS) was reduced 5 weeks after the completion of agomir treatment in 10–11-MO APP/PS1 mice. Representative images and quantification data from the hippocampus are shown. Scale bar, 100 μm. Data are presented as mean ± SEM, *n* = five mice each group. For (**g**) and (**h**), data were analyzed with one-way ANOVA

Amyloid plaques and neurofibrillary tangles are pathological hallmarks of AD [25]. The amyloid beta (Aβ) pathology in APP/PS1 mice occurs at around 6 months of age [22, 26]. To examine whether the improvement of cognitive functions by agomirs was correlated with changes in AD pathological markers, we first analyzed the changes of Aβ pathology in APP/PS1 mice. As shown in Additional file 1: Fig. S5a, agomir treatment did not change the total number of Aβ plaques in the hippocampus of APP/PS1 mice. However, we noticed that the hippocampal Aβ plaques existed in different forms, including the non-fibrillar diffuse plaques and classic cored plaques (also known as focal plaques) (Fig. 6a, and Additional file 1: Fig. S5a). Morphologically, the cored plaques showed a central aggregatin-positive amyloid core that facilitates Aβ aggregation [27], which was surrounded by a corona of non-fibrillar Aβ (Additional file 1: Fig. S5b). The cored plaques are positively correlated with AD neuropathology and cognitive dysfunction, while the diffuse plaques have no correlation with the severity of neuropathological changes in AD [28]. Consistent with this, the APP/PS1 mice showed a high load of classic cored Aβ plaques in the hippocampus (Fig. 6a, Additional file 1: Fig. S5a, left panel). Intriguingly, both forms of Aβ plaques were almost completely immunoreactive for CPE (Fig. 6a). Similar to the Aβ plaques, these CPE plaques also exhibited at least two distinct morphologies, which we named diffuse and cored CPE plaques.

Then, an in-depth immunostaining characterization of both cored and diffuse CPE plaques was performed in the hippocampus of APP/PS1 mice. Comparison of adjacent sections that immunoreacted either with CPE or aggregatin revealed that similar to the classic Aβ cored plaque (Additional file 1: Fig. S5b), CPE was observed as a small outer ring surrounding the aggregatin-positive center in the cored CPE plaque (Fig. 6b, left panel). In addition, cored CPE plaques were also immunoreactive for ApoE, hyperphosphorylated Tau (pTau, stained by pTau-S396 and AT8), mBDNF, proBDNF and Secretogranin III (Sg III, a secretory sorting receptor [29]) (Fig. 6b), although the degree of co-localization varied. Interestingly, the ApoE and AT8 immunoreactivity was observed more in the center than in the periphery of the CPE plaque, while the staining of pTau-S396, mBDNF, proBDNF and Sg III was more found in the corona of the CPE plaque (Fig. 6b). The diffuse CPE plaques in the APP/PS1 mice were only partially immunoreactive for ApoE, AT8, mBDNF, proBDNF and Sg III (Additional file 1: Fig. S5c).

We also examined endogenous CPE plaques in the WT mice of different ages. Quite different from that observed in the APP/PS1 mice, the CPE plaques in the WT mice showed a puncta appearance, and were named “CPE puncta”. These CPE puncta were apparent in the hippocampus of WT mice as early as 8 months of age. The number of CPE puncta increased age-dependently in the WT mice, and did not change much after 12 MO (Additional file 1: Fig. S5d). Noteworthy, cored CPE puncta were only found in even older mice (18 MO or older). Likewise, the CPE puncta in aged WT mice were found to be immunoreactive for aggregatin, ApoE, Aβ and mBDNF (Additional file 1: Fig. S5e).

BDNF has been shown to be associated with amyloid deposition in human AD [30, 31] as well as in AD mice [32]. In APP/PS1 mice, a similar upregulation of mBDNF was observed in CPE-plaque-associated glial cells, which was local rather than global (Fig. 6c). By immunostaining of these CPE plaques with Aβ and mBDNF, we found that the staining pattern of mBDNF in the cored CPE plaques was different from that in the diffuse CPE plaques: not much or no mBDNF staining was seen around the diffuse CPE/Aβ plaques (Fig. 6c, white arrows). For the cored CPE plaques, mBDNF was seen more in the periphery of plaques with small cores (Fig. 6d, top panel), and mostly in close proximity to (Fig. 6d, middle panel) or even within the corona of CPE plaques with larger cores (Fig. 6d, bottom panel). These results support the plaque-associated mBDNF upregulation in AD. Considering the possible role of CPE in the production of mBDNF [7] and its aggregation ability [33], we speculate that CPE may play a role in the plaque-associated mBDNF upregulation.

Recently, BDNF has been reported to promote the activation of microglia [34], further supporting its involvement in the inflammatory process [35, 36]. We next examined whether the plaque-associated mBDNF upregulation affects microglial activation. We found that the CPE plaques were surrounded by clusters of activated microglia (Iba1^+^) (Fig. 6e). In APP/PS1 mice, cored CPE plaques were co-localized with Iba1 to a certain degree (Fig. 6e). Of note, the cell bodies of amoeboid microglia, which show typical characteristics of phagocytosis [37, 38], were more frequently in close vicinity to the cores of cored CPE plaques than around diffuse CPE plaques (Fig. 6e, f). Similar patterns were observed in old WT mice (Additional file 1: Fig. 5f). Moreover, consistent with the local enrichment of mBDNF around the plaques, the clustering of Iba1-positive microglia was also puncta/plaque-associated, and it appeared that the bigger the plaque cores, the more the microglial cell bodies around the plaque cores (Fig. 6e). Together, these results suggest that these activated amoeboid microglia may have a higher potential for the clearance of cored plaques relative to diffuse plaques.

As CPE co-localized with Iba1 in the cored CPE plaques which are frequently surrounded by increased microglial phagocytosis, and based on the fact that CPE plaques appear concurrently with amyloid plaques, we suspected that both m10- and m37-agomirs might affect the load of cored plaques in APP/PS1 mice. Indeed, we found that the number of cored CPE plaques in the hippocampus of m10-/m37-agomir-treated APP/PS1 mice was significantly decreased compared with that in the NC-agomir group, although the number of total CPE plaques showed no change (Fig. 6g). This indicates that these agomirs could efficiently and specifically alleviate cored amyloid plaques in the hippocampus. Furthermore, we also examined the amount of fibrillar plaques (Aβ-sheet deposits) in the hippocampus using the ThS staining method. The positive area for Aβ aggregates in the hippocampus and the number of plaques per hippocampal area of m10-/m37-agomir-treated mice were significantly reduced relative to the NC-agomir-treated mice (Fig. 6h). Together, these results suggest that agomirs could effectively ameliorate Aβ pathology in APP/PS1 mice.

### miRNA agomirs ameliorate other AD pathologies in APP/PS1 mice

**Fig. 7:**
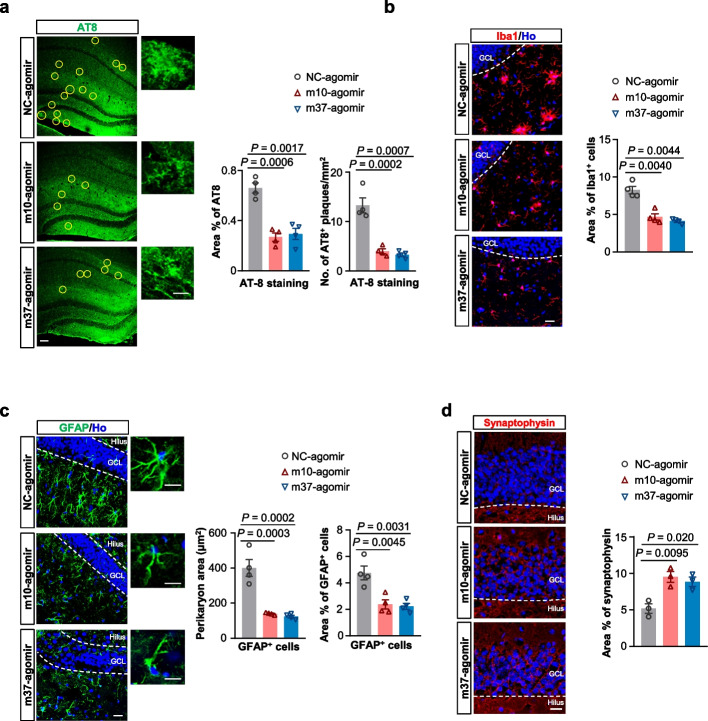
miRNA agomir treatment counteracts various AD pathologies in APP/PS1 mice. **a** Phosphorylated tau (antibody: AT8) levels are reduced 5 weeks after the completion of agomir treatment in 10–11-MO APP/PS1 mice. Representative images from the hippocampus are shown (left panel). Scale bar, 100 μm. Yellow circles indicate regions of AT8 positivity. Insets: high-magnification images of the representative AT8-positive plaques in each group. Scale bar, 20 μm. The area of AT8 reactivity and number of AT8 plaques per area were quantified within the hippocampus (right panel). Data are presented as mean ± SEM, *n* = four mice each group. **b** Agomir treatment reduces activated microglia 5 weeks after the completion of agomir treatment in the hippocampus of 10–11-MO APP/PS1 mice globally. Representative images from the hippocampus are shown. Scale bar, 20 μm. The area fraction positive for Iba1 was quantified within the hippocampus. *n* = four mice each group. Data are presented as mean ± SEM. **c** Agomir treatment reduces astrogliosis 5 weeks after the completion of agomir treatment in the hippocampus of 10–11-MO APP/PS1 mice. Representative images from the hippocampus are shown. Scale bar, 20 μm. High-magnification images of GFAP-positive hypertrophic glial cells are shown on the right. Scale bars, 10 μm. The average perikaryon area of glial cells and the percentage of the area of GFAP^+^ astrocytes were quantified within the hippocampus. Data are presented as mean ± SEM, *n* = four mice each group. **d** Representative images of synaptophysin expression 5 weeks after the completion of agomir treatment in the DG of 10–11-MO APP/PS1 mice (left panel). Scale bar, 20 µm. The quantification of the area fraction positive for synaptophysin within the DG is shown on the right. Data are presented as mean ± SEM, *n* = three mice each group. For (**a**-**d**), data were analyzed with one-way ANOVA

Another neuropathological hallmark of AD is the neurofibrillary pathology, which include neurofibrillary tangles (NFTs), neuropil threads and neuritic plaques [39]. Analysis of NFTs showed that m10-/m37-agomir significantly reduced the number of AT8-postive paired helical filament (PHF)-tau in comparison to the NC-agomir-treated APP/PS1 mice (Fig. 7a), suggesting that these agomirs could also affect the aggregation of tau protein.

As prominent AD pathological features, neuroinflammation and astrogliosis are closely associated with amyloid deposits in AD [40–42]. We then examined whether microglia and astrocyte distribution changed in the hippocampus upon agomir treatment, using Iba1 and GFAP as markers for microglia and astrocytes, respectively. Globally, APP/PS1 mice treated with m10-/m37-agomir exhibited less microgliosis compared to those treated with NC-agomir (Fig. 7b). Noteworthy, more hypertrophic glial cells were present in the NC-agomir-treated APP/PS1 mice. In addition, both the cytoplasm area surrounding the nucleus, namely the perikaryon area of astrocytes, and the area of GFAP^+^ astrocytes in the hippocampus were significantly decreased in size in m10-/m37-agomir-treated APP/PS1 mice compared to the NC-agomir-treated mice (Fig. 7c).

Synaptic loss is also thought to be core to the pathophysiology of AD [43] and synaptophysin expression is a marker for synaptic density. We then investigated the degree of synaptic loss in the agomir-treated APP/PS1 mice. The density of synaptophysin in the m10-/m37-agomir-treated APP/PS1 mice was significantly increased compared to the NC-agomir group (Fig. 7d), suggesting that these two agomirs may have protective effects against synaptic damage in AD.

Collectively, these results show that the two CPE-upregulating agomirs could exert protective effects in the hippocampus of APP/PS1 mice at multiple levels, suggesting that the agomir-induced boosting neurogenesis reduces the appearance of several AD hallmarks, thus influencing AD neuropathologies.

## Discussion

Hippocampus is one of the most affected areas in AD, and adult hippocampal neurogenesis persists but drops in aged and AD brains [3, 4]. In addition, dysfunction of BDNF signaling has also been shown to be involved in the pathogenesis of AD [44]. In the present study, we explored the role of CPE in adult hippocampal neurogenesis during aging and in AD. Importantly, we developed two agomirs, which were shown to upregulate CPE expression, promote adult hippocampal neurogenesis, improve newborn neuron dendritic development, rescue memory deficits and confer neuroprotection against various pathologies of AD in the hippocampal neurogenic niche of middle-aged APP/PS1 transgenic mice, thus showing great therapeutic potential to counteract AD.

Noteworthy, CPE showed overlapping expression with TrkB and mBDNF in NSCs of DG, which is consistent with our recent findings in the SVZ [7]. And in both cases, restoration of CPE has been shown to elevate mBDNF production and promote adult neurogenesis, suggesting that these functions of CPE are not specific to the SVZ but also in the SGZ. In support of this, CPE knockout (KO) or conditional KO mice also exhibit significant reduction of hippocampal neurogenesis compared to WT mice [45–47]. However, some discrepancies exist regarding the changes of proBDNF and mBDNF levels, which could possibly be due to the differences in experimental conditions, techniques used, and the age of animals [46]. In the present study, CPE did not influence the transcriptional level of BDNF, but rather enhanced the maturation of proBDNF to mBDNF, thereby increasing the level of mBDNF. Furthermore, in vitro data showed that CPE had similar effects on mBDNF level in cultured primary DG-NSCs (Fig. 2g) and SVZ-NSCs [7]. Further studies are needed to investigate the mechanisms underlying the CPE-induced elevation of mBDNF production in the hippocampus, in order to assess whether common mechanisms are shared with the effects of CPE on mBDNF production in the SVZ [7].

AD is generally believed to be caused by multiple pathogenic factors, such as Aβ, pTau and ApoE4. Although a link of CPE alterations with AD has been noted [29, 48], the role of CPE in the pathological progression of AD remains largely unknown. Here, we observed that the APP/PS1 mice show high-level and overlapping of CPE plaques and Aβ plaques in the hippocampus, both of which show at least two forms of morphology (cored and diffuse forms). Noteworthy, the cored CPE puncta only appeared at old ages (mostly > 18 MO). Given the fact that classic cored Aβ plaques are more associated with clinical dementia than the non-fibrillar type [28], these findings suggest the potential of CPE as a biomarker for the neuropathological changes under both physiological and pathological conditions.

The miRNA agomirs targeting CPE alleviated the AD-linked pathologies through two mechanisms. On one hand, the agomirs increased CPE expression to upregulate mBDNF production and promote adult neurogenesis in the hippocampus of APP/PS1 mice. CPE is present both intracellularly and in the extracellular space. Intracellular CPE is active at pH 5.5, within the internal pH range of secretory granules, and functions as an exopeptidase and a sorting receptor for targeting proBDNF to the regulated secretory pathway [49]. A number of studies have demonstrated that the impairment of neurogenesis and reduced BDNF expression in the hippocampus are closely associated with the pathogenesis of AD [50–54]. However, effective treatments targeting either BDNF or neurogenesis are limited. Notably, Choi et al. have shown that inducing adult hippocampal neurogenesis alone could not ameliorate cognitive function in AD mice unless both neurogenesis and mBDNF are increased [55]. This notion is supported by a recent study showing that miR-132, a potent regulator of adult hippocampal neurogenesis that promotes cognitive recovery in AD, can also positively affect BDNF levels [50]. These studies corroborate the significance of combining improvement of neurogenesis and BDNF as a potential therapeutic strategy for AD. In the present study, we found that the miRNA agomir-induced increase of CPE promoted mBDNF maturation, and significantly boosted adult neurogenesis possibly through upregulating the BDNF–TrkB signaling pathway and the FGF2-related pathway in both SVZ [7] and DG of aged or AD mice.

Although extracellular CPE no longer exhibits enzymatic activity at pH conditions around 7.0–7.3 thus failing to process proBDNF, it may function as a neuroprotective factor to ameliorate the AD-linked pathologies by interacting with human serotonin receptor HTR1E to activate the ERK–CREB signaling pathway, leading to the upregulation of mitochondrial prosurvival protein BCL2 [56]. A recent study showed that the secreted CPE can be internalized to lysosomes of neighboring cells in vitro [57], yet the probability and efficiency of extracellular CPE internalization to neuronal cells in the brain still require further investigation. The reason for accumulation of extracellular CPE within plaques in aged and AD brains remains unclear. We hypothesize that this might be linked to the fact that CPE can be efficiently secreted and is capable of self-aggregation [33]. There may be other mechanisms responsible for the extracellular secretion of CPE under pathological conditions. Immunostaining data showed that these CPE puncta were immunoreactive for ApoE and phosphorylated tau, both of which are prone to aggregate [58, 59]. Moreover, similar to aggregatin, these proteins were located more in the center of the cored CPE puncta. Based on these morphological and biochemical characteristics, we speculate that there may be a complex interplay among CPE expression, Aβ specific forms, Tau and ApoE aggregation, and other unknown factors responsible for this deposition formation, which deserves further investigation.

On the other hand, the two agomirs significantly attenuated the load of cored amyloid plaques in APP/PS1 mice. We hypothesized that CPE could effectively and specifically enhance the clearance of the cored amyloid plaques in the hippocampus of APP/PS1 mice, probably via activated local microglia which could phagocytose the existing plaques. How CPE leads to the activation of microglia remains undefined. Special attention should be paid to the possible link of the plaque-associated mBDNF upregulation and local microglial activation. BDNF is a crucial signaling molecule linking microglia and neurons. It has been shown that the expression and secretion of BDNF by neurons can suppress the widespread activation of hippocampal microglial cells [60]. The increased BDNF signaling inhibits aging-induced microglial activation through the TrkB–Erk–CREB pathway [61]. In our study, we also found that the agomir treatment ameliorated microgliosis in the hippocampus (Fig. 7b). However, several studies have shown that microglial cells express BDNF to selectively promote their own proliferation and function [62, 63], which is consistent with the local upregulation of mBDNF in glial cells and microglial activation surrounding the cored CPE-plaques. Although the mechanism of the dynamic changes of cored CPE plaques with age or in AD remains unclear, these interesting findings provide new insights into the association of CPE with AD. In support of this, recent research has shown that overexpression of CPE via viral-(AAV) delivery in the hippocampus of early-age AD mice prevents AD progression through distinct mechanisms. In that study, CPE inhibited hippocampal neurodegeneration, decreased APP mRNA expression through downregulating expression of transcription factors *Sp1* and *Hsf-1*, and significantly reduced insoluble Aβ levels and tau hyperphosphorylation [64]. Furthermore, CPE protects neurons from cell death in the hippocampus [47] and facilitates mitophagy through reduced expression of the mitophagy inhibitor PLIN4 in AD mice [64], all of which could potentially aid in alleviation of AD-linked pathologies.

In line with these findings, here, we show that restoring the CPE expression in situ by agomirs could promote mBDNF generation, boost hippocampal neurogenesis, increase dendritic length and complexity in the DG, and more importantly, ameliorate memory deficits in APP/PS1 mice by exerting various beneficial effects on AD-linked pathologies at multiple levels. Of note, replenishment of CPE expression by a non-invasive approach via the intranasal delivery of agomirs is sufficient to improve the behavioral and neurocognitive functions in APP/PS1 mice, making it particularly useful for clinical development into AD drugs. Moreover, considering that chemically modulated miRNA agomirs have higher stability and can be delivered easily [65–67], CPE targeting by agomirs is a potential novel therapeutic candidate for AD and other neurodegenerative disorders.

## Conclusions

In summary, our data, together with the previous study [7], show that CPE can regulate adult neurogenesis in both DG and SVZ, probably via the common BDNF–TrkB signaling pathway. The two CPE-upregulating agomirs would have considerable clinical implications for AD as they could increase both adult neurogenesis and mBDNF levels in the hippocampus, and more importantly, could reverse the behavioral and neurocognitive declines in APP/PS1 mice even delivered via a non-invasive intranasal instillation approach. Therefore, these findings support the prospect of developing miRNA agomirs targeting CPE as biopharmaceuticals for the treatment of AD in the future.

### Supplementary Information

**Additional file 1: Fig. S1.** In vivo characterization of TrkB, mBDNF and CPE expression in the DG. **Fig. S2.** Restoration of CPE level promotes hippocampal neurogenesis. **Fig. S3.** Screened miRNAs upregulate CPE expression both in vitro and in vivo. **Fig. S4.** CPE-upregulating miRNA agomirs promote mBDNF and FGF2 expression, and rescue memory deficits in APP/PS1 mice. **Fig. S5.** Characterization of Aβ and CPE plaques/puncta in the hippocampus of APP/PS1 and aged WT mice.

## Data Availability

All data generated or analyzed during this study are included in this published article and its supplementary information files.

## Abbreviations

Aβ: Amyloid beta
AD: Alzheimer’s disease
ANOVA: Analysis of variance
APP/PS1: APPswe/PSEN1ΔE9 double transgenic
BDNF: Brain-derived neurotrophic factor
CPE: Carboxypeptidase E
DCX: Doublecortin
DG: Dentate gyrus
GCL: Granular cell layer
ICV: Intracerebroventricular
IPCs: Intermediate progenitor cells
mBDNF: Mature BDNF
miRNA: MicroRNA
ML: Molecular layer
MWM: Morris water maze
NSCs: Neural stem cells
NBs: Neuroblasts
PHF: Paired helical filament
Sg III: Secretogranin III
SVZ: Subventricular zone
SGZ: Subgranular zone
ThS: Thioflavin S
3′UTR: 3′-Untranslated region
WT: Wild-type
