# Supplementary material for: Agomirs upregulating carboxypeptidase E expression rescue hippocampal neurogenesis and memory deficits in Alzheimer’s disease
Source: Transl Neurodegener. 2024 Apr 26;13:24. doi: 10.1186/s40035-024-00414-z (PMC11046780; doi:10.1186/s40035-024-00414-z)
Supplement: Supplementary file 1 — Additional file 1: Fig. S1. In vivo characterization of TrkB, mBDNF and CPE expression in the DG. Fig. S2. Restoration of CPE level promotes hippocampal neurogenesis. Fig. S3. Screened miRNAs upregulate CPE expression both in vitro and in vivo. Fig. S4. CPE-upregulating miRNA agomirs promote mBDNF and FGF2 expression, and rescue memory deficits in APP/PS1 mice. Fig. S5. Characterization of Aβ and CPE plaques/puncta in the hippocampus of APP/PS1 and aged WT mice. [file 40035_2024_414_MOESM1_ESM.pdf]

Figure S1

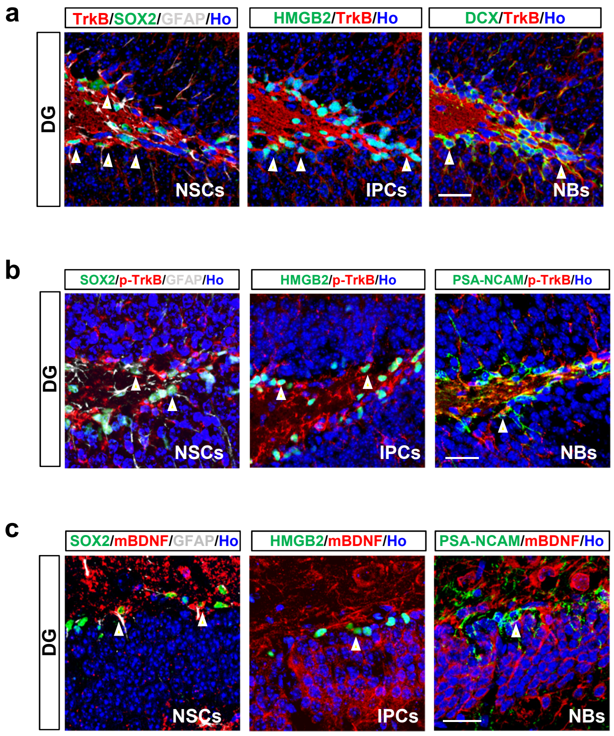

Fig. S1. ***In vivo*** **characterization of TrkB, mBDNF and CPE expression in the DG.** **a-c** Characterization of TrkB- **(a)** or p-TrkB- **(b)** or mBDNF- **(c)** positive NSCs in the DG of 2 MO WT mice. White arrowheads indicate SOX2+GFAP+ NSCs, HMGB2+ IPCs and DCX/PSA-NCAM+ NBs in the DG. Ho, Hoechst. Scale bars, 20 µm.

Figure S2

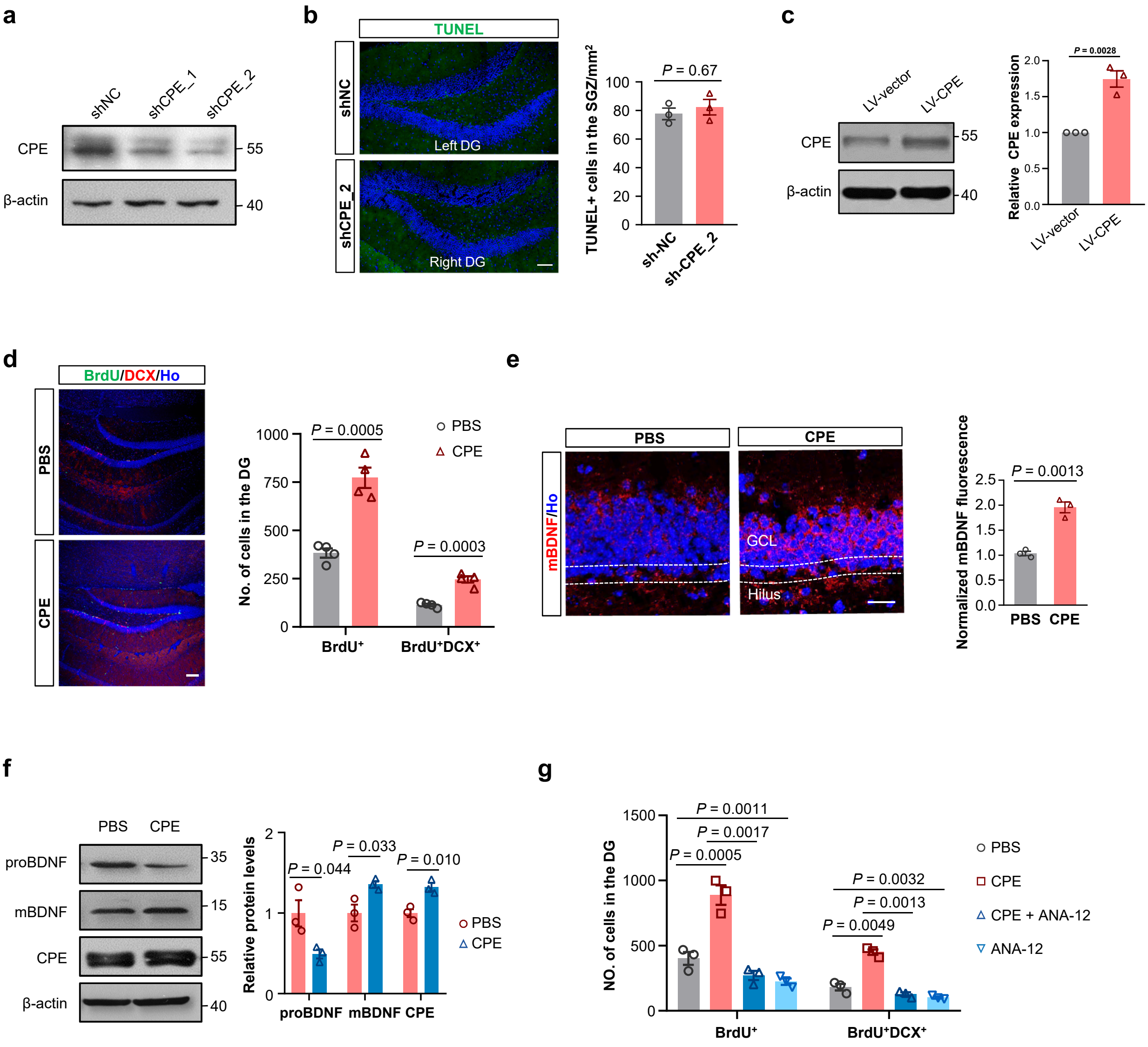
a
shNC
shCPE_1
shCPE_2
CPE
β-actin
55
40
b
TUNEL
shNC
shCPE_2
Left DG
Right DG
TUNEL+ cells in the SGZ/mm²
P = 0.67
sh-NC
sh-CPE_2
c
LV-vector
LV-CPE
CPE
β-actin
55
40
Relative CPE expression
P = 0.0028
d
BrdU/DCX/Ho
PBS
CPE
No. of cells in the DG
P = 0.0005
P = 0.0003
BrdU⁺
BrdU⁺DCX⁺
e
mBDNF/Ho
PBS
CPE
GCL
Hilus
Normalized mBDNF fluorescence
P = 0.0013
f
PBS
CPE
proBDNF
mBDNF
CPE
β-actin
35
15
55
40
Relative protein levels
P = 0.044
P = 0.033
P = 0.010
proBDNF mBDNF CPE
g
NO. of cells in the DG
P = 0.0011
P = 0.0017
P = 0.0005
P = 0.0032
P = 0.0013
P = 0.0049
BrdU⁺
BrdU⁺DCX⁺
PBS
CPE
CPE + ANA-12
ANA-12

Fig. S2. Restoration of CPE level promotes hippocampal neurogenesis. a Western blot analysis of the efficacy of the shRNA against mouse CPE in MEF cells. **b** Representative images of TUNEL labeling in the DG of middle-aged WT mice one week after lentiviruses expressing shNC or shCPE_2 injection. Scale bar, 20 μm. Quantification of TUNEL+ cells in the SGZ is shown on the right. Data are represented as mean ± SEM, n = three mice each group. One-way ANOVA. **c** Western blotting analysis of CPE expression levels in the hippocampus of middle-aged WT mice one week after lentiviruses expressing NC (LV-NC) or wild-type CPE (LV-CPE) injection. β-actin is used as a loading control. Relative quantification of protein levels is shown on the right. Data are represented as mean ± SEM from three mice each group. Two-tailed unpaired Student's t test. **d** Representative images of BrdU and DCX double-labelled newly generated neurons in the DG of middle-aged WT mice one week after PBS or CPE icv infusion. Scale bar, 100 μm. Quantification of BrdU+ and BrdU+DCX+ cells in the SGZ is shown on the right. Data are represented as mean ± SEM, n = four mice each group. Two-tailed Student's t test. **e** Representative images of mBDNF expression in the DG of middle-aged WT mice one week after PBS or CPE infusion. Scale bar, 50 μm. Normalized mBDNF fluorescence intensity in the DG is shown on the right. Data are represented as mean ± SEM, n = three mice each group. Two-tailed Student's t test. **f** Western blotting analyses of proteins extracted from the mixed tissues of hippocampus from three middle-aged WT mice one week after PBS or CPE icv infusion. β-actin is used as a loading control. Relative quantification of protein levels is shown on the right. Data are represented as mean ± SEM from three mice each group. Two-tailed Student's t test. **g** Quantification of BrdU+ and BrdU+DCX+ cells in the DG of CPE-infused middle-aged WT mice with or without the TrkB antagonist ANA-12 treatment. n = three mice each group. One-way ANOVA.

Figure S3

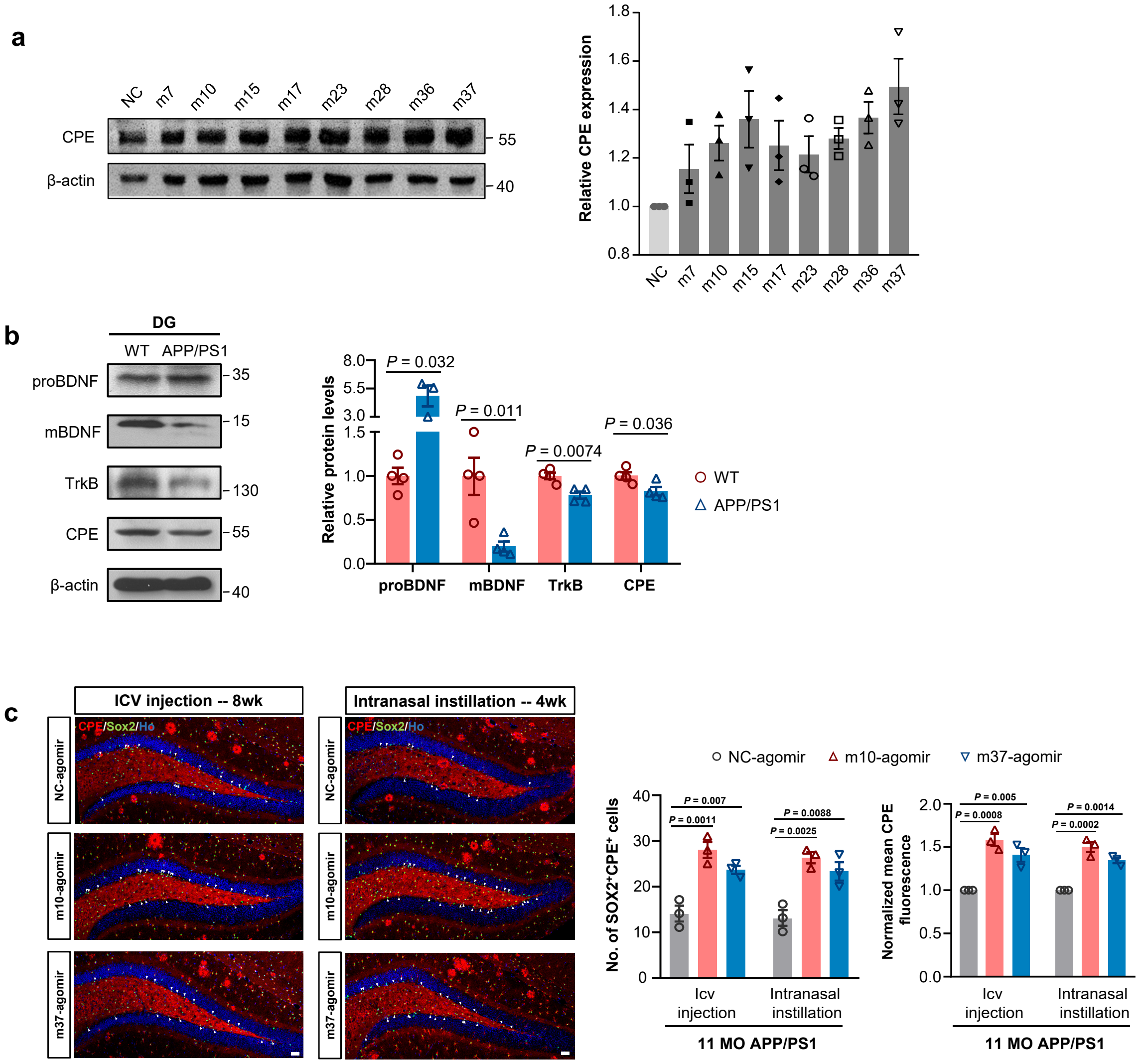

Fig. S3. Screened miRNAs upregulate CPE expression both *in vitro* and *in vivo.* a Western blotting analyses of CPE expression in N2a cells 30 h after miRNA mimics transfection. Relative quantification of Western blotting analysis of CPE levels is shown on the right. β-actin is used as a loading control. Relative quantification of CPE levels is shown on the right. Data are represented as mean ± SEM from three independent experiments. **b** Western blotting analyses of proteins extracted from the mixed tissues of hippocampus from three 9 MO APP/PS1 mice and three age-matched WT mice. β-actin is used as a loading control. Relative quantification of protein levels is shown on the right. Data are represented as mean ± SEM from three mice each group. Two-tailed Student's t-test. **c** Representative images, quantification of SOX2+CPE+ cells in the SGZ and normalized fluorescence intensity of CPE expression in the DG of the 11 MO APP/PS1 mice eight weeks after miRNA agomirs ICV injections or four weeks after the 30-day agomirs treatment intranasally. Ho, Hoechst. Scale bars, 20 μm. Data are represented as mean ± SEM, n = three mice each group. Data were analyzed with one-way ANOVA.

Figure S4

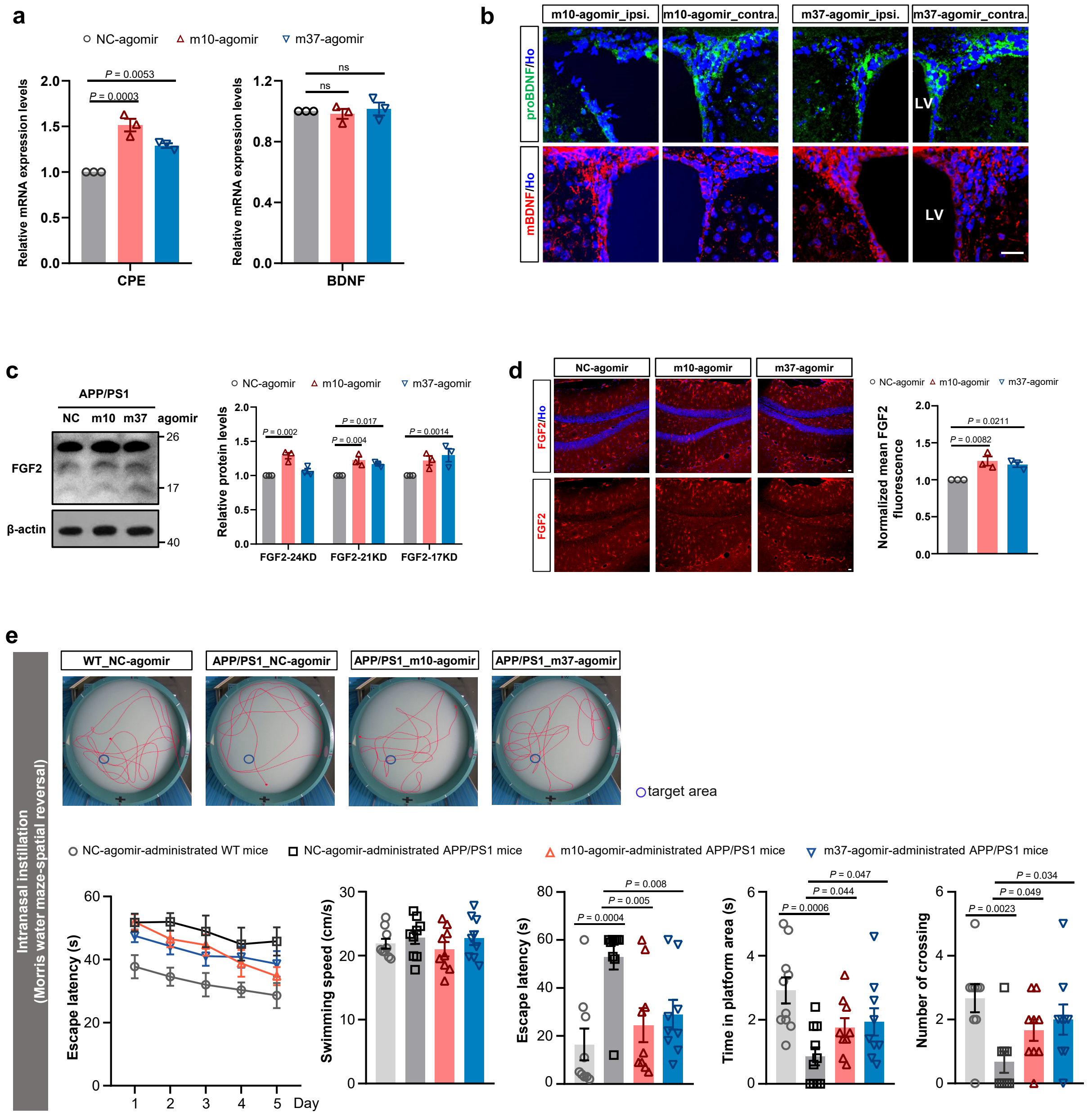

a
NC-agomir
m10-agomir
m37-agomir
Relative mRNA expression levels
P = 0.0053
P = 0.0003
CPE
ns
BDNF
b
m10-agomir_ipsi.
m10-agomir_contra.
m37-agomir_ipsi.
m37-agomir_contra.
proBDNF/Ho
mBDNF/Ho
LV
c
APP/PS1
NC m10 m37 agomir
FGF2
β-actin
26
17
40
Relative protein levels
P = 0.002
P = 0.017
P = 0.004
P = 0.0014
FGF2-24KD
FGF2-21KD
FGF2-17KD
d
NC-agomir
m10-agomir
m37-agomir
FGF2/Ho
FGF2
Normalized mean FGF2 fluorescence
P = 0.0211
P = 0.0082
e
Intranasal instillation (Morris water maze-spatial reversal)
WT_NC-agomir
APP/PS1_NC-agomir
APP/PS1_m10-agomir
APP/PS1_m37-agomir
target area
NC-agomir-administrated WT mice
NC-agomir-administrated APP/PS1 mice
m10-agomir-administrated APP/PS1 mice
m37-agomir-administrated APP/PS1 mice
Escape latency (s)
Day
Swimming speed (cm/s)
Escape latency (s)
P = 0.008
P = 0.005
P = 0.0004
Time in platform area (s)
P = 0.047
P = 0.044
P = 0.0006
Number of crossing
P = 0.034
P = 0.049
P = 0.0023

Fig. S4. CPE-upregulating miRNA agomirs promote mBDNF and FGF2 expression, and rescue memory deficits in APP/PS1 mice. a The mRNA expression levels of CPE and BDNF in the hippocampus of 9 MO APP/PS1 mice two weeks after miRNA agomirs icv injection. GAPDH was used as the control. Data are represented as mean ± SEM, n = three mice each group. Data were analyzed with one-way ANOVA. **b** Representative images of proBDNF and mBDNF staining in the SVZ areas from one single 9 MO APP/PS1 mice two weeks after miRNA agomirs icv injection. Scale bar, 50 μm. ipsi., ipsilateral; contra., contralateral. **c** Western blotting analysis of FGF2 expression levels in the hippocampus of 9 MO APP/PS1 mice two weeks after miRNA agomirs icv injection. **d** Representative images and normalized fluorescence intensity of FGF2 expression in the DG of 9 MO APP/PS1 mice two weeks after miRNA agomirs injections. Ho, Hoechst. Scale bars, 20 μm. **e** Rescue of behavioral deficit in the spatial reversal of MWM test in 3-4 weeks after the completion of the 30-day treatment intranasally to APP/PS1 mice. Latency to reach the platform in the acquisition phase, representative tracing pathway, swimming speed, escape latency to reach the original platform location, time in platform area, and number of crossings during the probe trial are presented. Data are represented as mean ± SEM, n = 7 – 11 mice each group. Data were analyzed with one-way ANOVA.

Figure S5

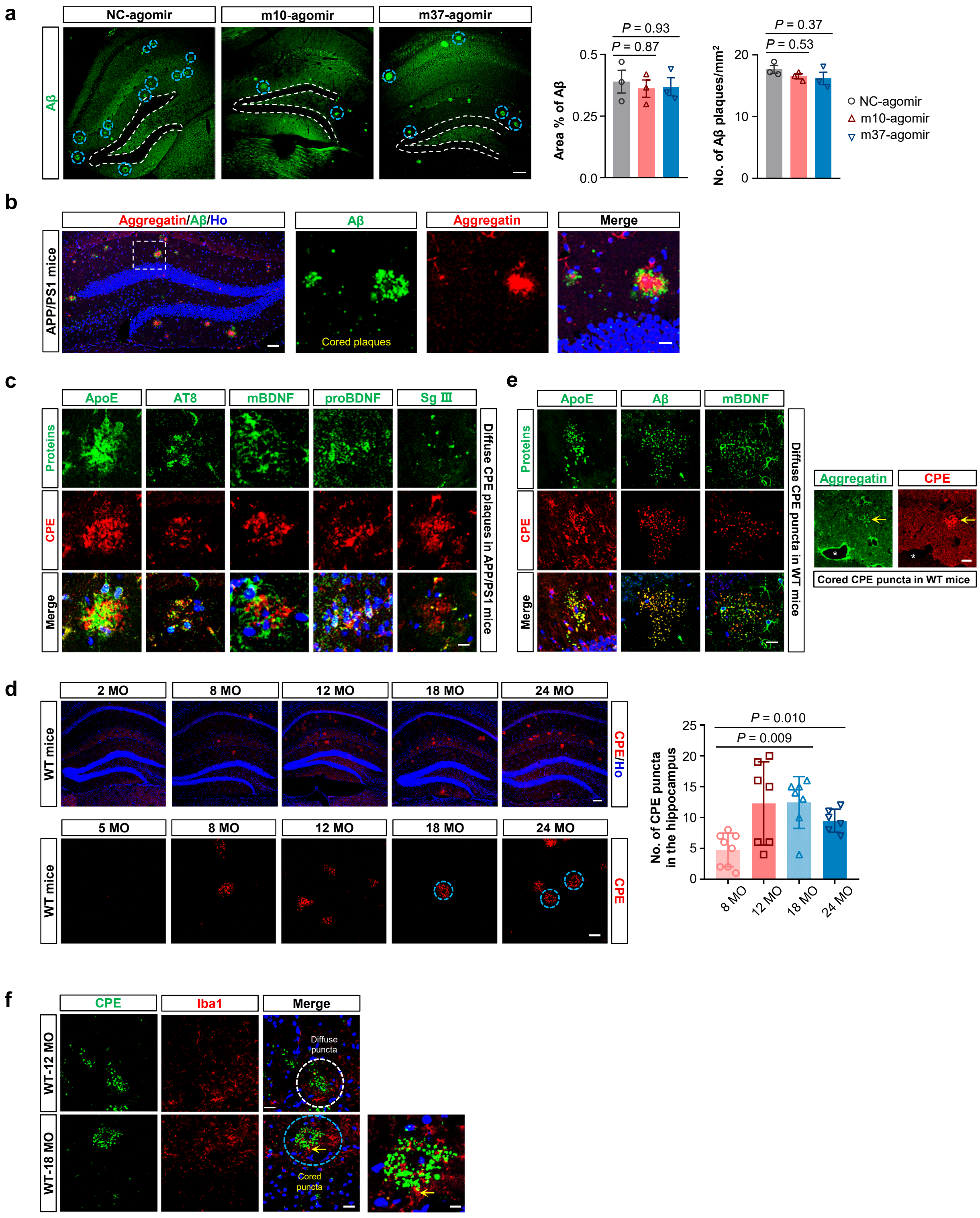
a
NC-agomir
m10-agomir
m37-agomir
Aβ
P = 0.93
P = 0.87
Area % of Aβ
0.5
0.25
0.0
P = 0.37
P = 0.53
No. of Aβ plaques/mm²
20
10
0
NC-agomir
m10-agomir
m37-agomir
b
Aggregatin/Aβ/Ho
Aβ
Aggregatin
Merge
APP/PS1 mice
Cored plaques
c
ApoE
AT8
mBDNF
proBDNF
Sg Ⅲ
Proteins
CPE
Merge
Diffuse CPE plaques in APP/PS1 mice
e
ApoE
Aβ
mBDNF
Proteins
CPE
Merge
Diffuse CPE puncta in WT mice
Aggregatin
CPE
Cored CPE puncta in WT mice
d
2 MO
8 MO
12 MO
18 MO
24 MO
WT mice
CPE/Ho
5 MO
8 MO
12 MO
18 MO
24 MO
WT mice
CPE
P = 0.010
P = 0.009
No. of CPE puncta in the hippocampus
25
20
15
10
5
0
8 MO
12 MO
18 MO
24 MO
f
CPE
Iba1
Merge
WT-12 MO
Diffuse puncta
WT-18 MO
Cored puncta

Fig. S5. Characterization of Aβ and CPE plaques/puncta in the hippocampus of APP/PS1 and aged WT mice. a Representative images and quantifications of the area fraction positive to 6E10 and the number of total Aβ plaques per area five weeks after the completion of agomirs treatment in the DG of 10-11 MO APP/PS1 mice are shown. Scale bar, 100 μm. Blue dotted circles indicate classic cored plaques. Data are represented as mean ± SEM, n = three mice each group. One-way ANOVA. **b** The co-localization of aggregatin and Aβ in the hippocampus of 10-11 MO APP/PS1 mice. Scale bar, 100 μm. High magnification images from the box area in the left panel are shown on the right. Scale bar, 20 μm. **c** Various protein immunoreactivity of the diffuse CPE plaques in the hippocampus of 10-11 MO APP/PS1 mice. Scale bar, 20 μm. **d** Cored CPE puncta seem to be appeared in the hippocampus of WT mice older than 18 MO while diffuse CPE puncta start to appear as early as 8 MO in WT mice during aging. High magnification images were shown on the bottom. The quantification of the number of CPE puncta per area within the hippocampus during aging were shown on the right. Data are represented as mean ± SEM, n = six to eight mice each group. One-way ANOVA. **e** ApoE, Aβ and mBDNF immunoreactivity of the diffuse CPE puncta in the hippocampus of WT mice (12 MO) (Left); On the right, representative images showed immunostaining for cored CPE puncta and aggregatin (yellow arrows) in adjacent sections (denoted by white asterisks) of hippocampus from 18 MO of WT mice. Scale bars, 20 μm. **f** CPE puncta in the hippocampus of aged (12 and 18 MO) WT mice were associated with clusters of activated (Iba-1-positive) microglial. High magnification was shown on the right. Yellow arrow indicates microglia cell body. Blue dotted circles represent cored puncta while white dotted circles represent diffuse puncta. Scale bar, 20 μm.
